# Chemical and chemoenzymatic routes to bridged homoarabinofuranosylpyrimidines: Bicyclic AZT analogues

**DOI:** 10.3762/bjoc.18.10

**Published:** 2022-01-11

**Authors:** Sandeep Kumar, Jyotirmoy Maity, Banty Kumar, Sumit Kumar, Ashok K Prasad

**Affiliations:** 1 Bioorganic Laboratory, Department of Chemistry, University of Delhi, Delhi- 110 007, India; 2 Department of Chemistry, St. Stephen’s College, University of Delhi, Delhi- 110 007, India; 3 Department of Chemistry, Rajdhani College, University of Delhi, Delhi- 110 015, India

**Keywords:** bicyclic AZT analogues, bridged homoarabinofuranosylpyrimidine nucleosides, chemical pathway, Lipozyme^®^ TL IM, regioselective enzymatic acetylation

## Abstract

Conformationally restricted diastereomeric homoarabinofuranosylpyrimidines (AZT analogue), i.e., (5′*R*)-3′-azido-3′-deoxy-2′-*O*,5′-*C*-bridged-β-ᴅ-homoarabinofuranosylthymine and -uracil had been synthesized starting from diacetone ᴅ-glucofuranose following chemoenzymatic and chemical routes in 34–35% and 24–25% overall yields, respectively. The quantitative and diastereoselective acetylation of primary hydroxy over two secondary hydroxy groups present in the key nucleoside precursor was mediated with Lipozyme^®^ TL IM in 2-methyltetrahydrofuran following a chemoenzymatic pathway. Whereas, the protection of the primary hydroxy over the lone secondary hydroxy group in the key azido sugar precursor was achieved using bulky *tert*-butyldiphenylsilyl chloride (TBDPS-Cl) in pyridine in 92% yield following a chemical synthetic pathway. The chemoenzymatic method was found to be superior over the chemical method in respect of the number of synthetic steps and overall yield of the final product.

## Introduction

**Figure 1:**
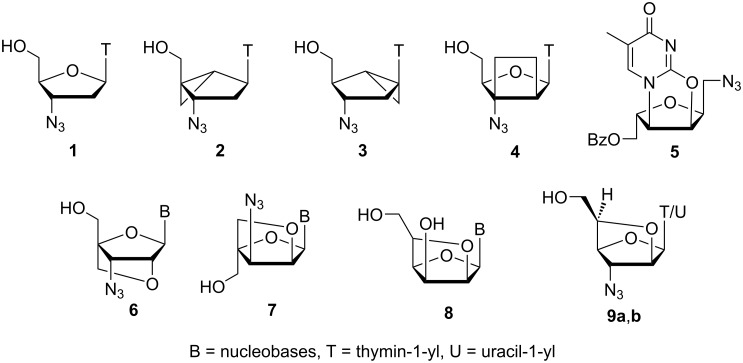
Structure of AZT and representative related bicyclic nucleosides.

In the last few decades, modification of nucleoside/nucleotide analogues has been a field of keen interest to researchers due to their therapeutic properties for treatment of cancer, viral and microbial infections [1–9]. The very first cytotoxic chemotherapeutic agents used for the treatment of cancer were nucleoside analogues and nucleobases [10]. Azidothymidine (**1**, AZT) was the first approved drug for the treatment of human immunodeficiency virus (HIV) [11–12]. Subsequently, a large number of sugar modified nucleosides, such as ddC (zalcitabine) [13–14], ddI (didanosine) [15], d4T (stavudine) [16–17], 3TC (lamivudine) [18–19] and AZT analogues were synthesized and evaluated towards inhibition of HIV reverse transcriptase (HIV RT). However, the toxic side effects associated with these molecules and development of drug-resistant viruses eventually brought about the need for newly designed and improved anti-HIV drugs with respect to their improved pharmacological properties [20]. With an idea to keep the presence of azide functionality and to introduce structural rigidity, Marquez et al. [21] developed a methodology for the synthesis of azido-carbobicyclic nucleosides **2** and **3**, where compound **2** showed activity similar to that of AZT while compound **3** was devoid of any type of activity (Figure 1). Nielsen et al. [22] synthesized the conformationally restricted nucleoside **4**, which was devoid of any anti-HIV activity. Further, Zhang et al. [23] synthesized the tricyclic azido-isonucleoside analogue **5** and Imanishi et al. [24] synthesized the azido-nucleoside **6** with a conformationally restricted sugar moiety. Recently, we have developed a greener and efficient chemoenzymatic synthetic methodology for the synthesis of conformationally restricted bicyclic azido-nucleosides **6** [25] and **7** [26], and bicyclic homonucleosides **8** [27]. Herein, we report an efficient chemo-enzymatic and chemical route to synthesize two novel conformationally restricted azido-homoarabino nucleosides **9a**,**b** in excellent overall yields (Figure 1). To the best of our knowledge, this is the first report of a synthesis of azido-bicyclic β-ᴅ-homoarabinofuranosyl nucleosides, i.e., bicyclic homonucleoside analogues of AZT.

## Results and Discussion

**Scheme 1:**
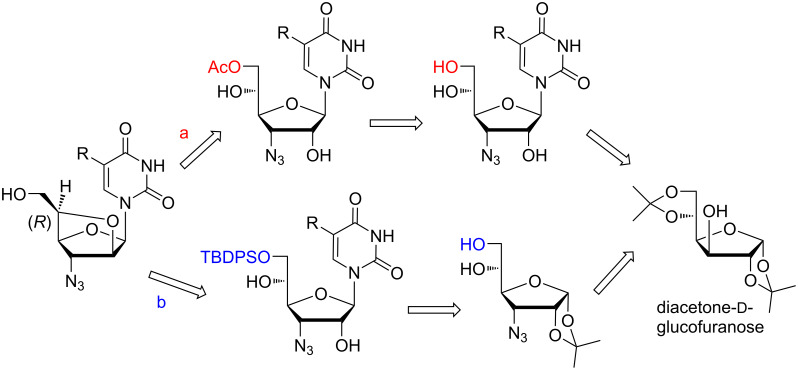
Retrosynthetic routes for the synthesis of (5′*R*)-3′-azido-3′-deoxy-2′-*O*,5′-*C*-bridged-β-ᴅ-homoarabinofuranosylpyrimidines following a chemoenzymatic pathway (a) and a chemical pathway (b).

It was envisioned to synthesize targeted bridged homoarabinofuranosyl nucleosides starting from diacetone-ᴅ-glucofuranose following chemoenzymatic and chemical pathways (Scheme 1).

**Scheme 2:**
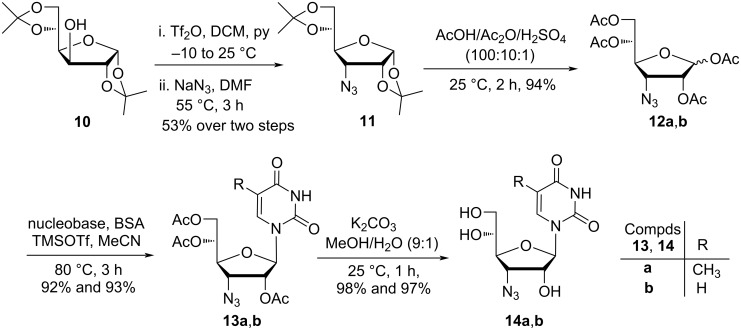
Synthesis of 3′-azido-3′-deoxy-β-ᴅ-allofuranosylthymine (**14a**) and -uracil (**14b**).

Thus, in the chemoenzymatic approach, the synthesis of targeted bicyclic homonucleosides started with the conversion of diacetone ᴅ-glucofuranose **10** into 3-azido-3-deoxydiacetone-ᴅ-allofuranoside **11** following a literature procedure in 53% yield [28]. Azidofuranoside **11** on acetolysis with acetic acid/acetic anhydride/sulfuric acid (100:10:0.1) at room temperature afforded an anomeric mixture of 1,2,5,6-tetra-*O*-acetyl-3-azido-3-deoxy-α,β-ᴅ-allofuranose (**12a**,**b**), which on Vorbrüggen base coupling [29] with thymine and uracil in the presence of *N*,*O*-bis(trimethylsilyl)acetamide (BSA) and trimethylsilyl trifluoromethanesulfonate (TMSOTf) afforded nucleosides **13a**,**b**. Further, deacetylation of the triacetylated azidoallofuranosyl nucleosides **13a**,**b** with K_2_CO_3_ in methanol/water led to the formation of 3′-azido-3′-deoxy-β-ᴅ-allofuranosyl nucleosides **14a**,**b** in 85% overall yield starting from compound **11**, respectively (Scheme 2).

**Scheme 3:**
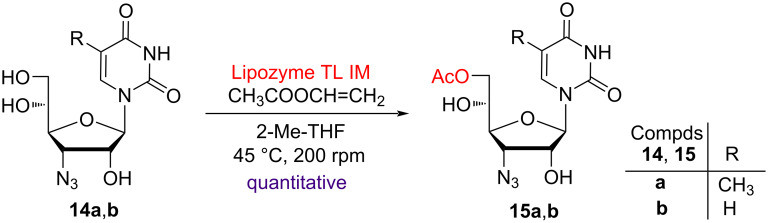
Biocatalytic acetylation of the primary hydroxy group of nucleosides **14a**,**b**.

The use of lipase as biocatalyst was employed for the selective acetylation of the primary hydroxy group present in trihydroxy nucleosides **14a**,**b**. This led to the screening of two different lipases, viz *Candida antarctica* lipase-B (CAL-B) immobilized on polyacrylate (Lewatit), commonly known as Novozyme^®^ 435 and *Thermomyces lanuginosus* lipase immobilized on silica, commonly known as Lipozyme^®^ TL IM in different organic solvents, such as THF, acetonitrile, toluene, acetone, DIPE and 2-Me-THF. Vinyl acetate was used as acetyl donor at temperatures ranging from 25–45 °C in an incubator shaker at 200 rpm. Lipozyme^®^ TL IM (10 wt % of the substrate) in 2-Me-THF at 40 °C was found to be the most suitable solvent for exclusive acetylation of the primary hydroxy group present in compounds **14a**,**b** in quantitative yields (Scheme 3). Further, the reusability of the recovered Lipozyme^®^ TL IM was also checked and it was found that the lipase could be used up to seven cycles of selective acetylation of 3′-azido-3′-deoxy-β-ᴅ-allofuranosyl nucleosides **14a**,**b** without noticeable loss of regioselectivity and efficiency. Thus, trihydroxy nucleosides **14a**,**b** were treated in 2-Me-THF with Lipozyme^®^ TL IM (10 wt % of the substrate nucleoside) and vinyl acetate at 40 °C and 200 rpm in an incubator shaker to afford the monoacetylated nucleosides **15a**,**b** in quantitative yields in 2 h (Scheme 3).

**Scheme 4:**
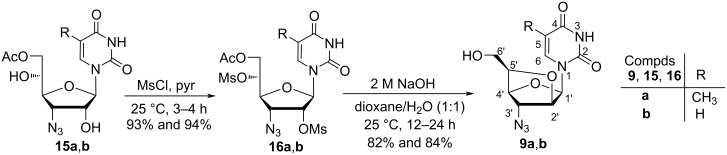
Synthesis of (5*'R*)-3′-azido-3′-deoxy-2′-*O*,5′-*C*-bridged-β-ᴅ-homoarabinofuranosyl nucleosides **9a**,**b**.

The monoacetylated nucleosides **15a**,**b** were mesylated using mesyl chloride in pyridine to afford dimesylated nucleosides **16a**,**b** in 93 and 94% yields, respectively. The reaction of nucleosides **16a**,**b** with NaOH in dioxane/water (1:1) underwent a cascade reaction pathway to form **9a**,**b** in 82 and 84% yields, respectively (Scheme 4). The overall yields for the synthesis of nucleosides **9a**,**b** starting from diacetone ᴅ-glucofuranose were found to be 33.9 and 35.1%, respectively.

In our earlier research work, bridged homolyxofuranosylpyrimidines were synthesized following a chemoenzymatic methodology where biocatalyst Novozyme 435 was used for regioselective acetylation of the primary hydroxy group present in 3′-*O*-benzyl-β-ᴅ-glucofuranosylpyrimidines [27]. In this article, regioselective acylation at the primary hydroxy group of 3′-azido-3′-deoxy-β-ᴅ-allofuranosylpyrimidines was carried out with the different biocatalyst Lipozyme TL IM using the same acylating agent, i.e*.*, vinyl acetate but in a different solvent, i.e., 2-Me-THF instead of THF and at a different temperature, i.e., 40 °C instead of 45 ^o^C as mentioned in the previous reaction conditions. It is noteworthy to mention that both of these biocatalytic reactions afforded the desired products in quantitative yield.

**Scheme 5:**
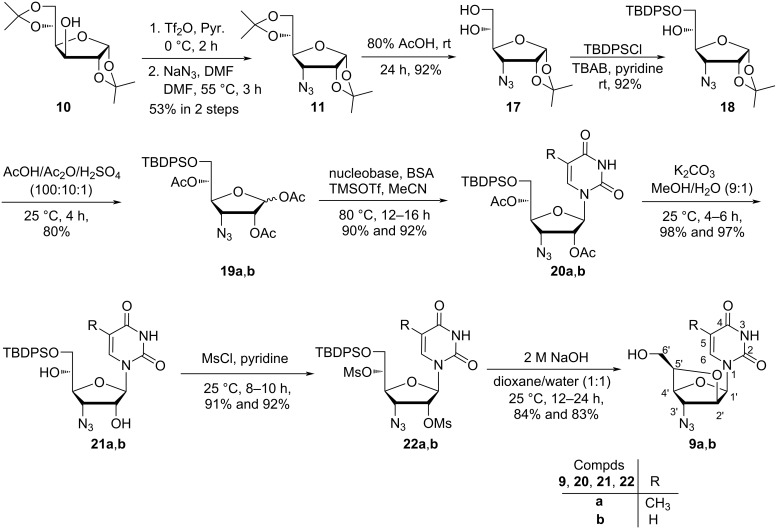
Chemical synthesis of nucleosides **9a**,**b** starting from diacetone ᴅ-glucofuranose.

At first, trihydroxy nucleoside **14b** was protected with TBDPS to carry out the synthesis of the target nucleoside monomer **9b** through a chemical pathway. However, the poor yield of this reaction compelled us to abandon the route as high solubility of the TBDPS-protected dihydroxy nucleoside in aqueous phase withstood its extraction in the organic phase during work up procedure. So, the classical chemical route to synthesize nucleosides **9a**,**b** started with the conversion of diacetone ᴅ-glucofuranose **10** to 3′-azido-3′-deoxy-β-ᴅ-allofuranoside **11**, which on acidification with 80% acetic acid gave furanoside diol **17**. The regioselective protection of the primary hydroxy group of diol **17** using TBDPS-Cl in pyridine at room temperature afforded TBDPS-protected furanoside **18**, which on acetolysis using AcOH/Ac_2_O/H_2_SO_4_ (100:10:0.1) afforded the anomeric mixture of coupling sugar **19a**,**b** in 80% yield. The Vorbrüggen coupling [29] of anomeric triacetylated sugars **19a**,**b** with thymine and uracil in the presence of BSA and TMSOTf in acetonitrile afforded nucleosides **20a**,**b**, respectively in high yields. The deacetylation of these nucleosides was carried out using K_2_CO_3_ in a solvent mixture of methanol and water (9:1) to afford nucleoside **21a**,**b**, which in turn on mesylation with mesyl chloride in pyridine afforded dimesylated nucleosides **22a**,**b**. Nucleosides **22a**,**b** on treatment with aqueous sodium hydroxide in a solvent mixture of dioxane and water (1:1) afforded 2′-*O*,5′-*C*-bridged-β-ᴅ-homoarabinofuranosyl nucleosides **9a**,**b** in 84 and 83% yields, respectively (Scheme 5). The overall yields of the synthesis of nucleosides **9a** and **9b** starting from diacetone ᴅ-glucofuranose following the classical chemical pathway were found to be 24.2 and 24.4%, respectively.

**Figure 2:**
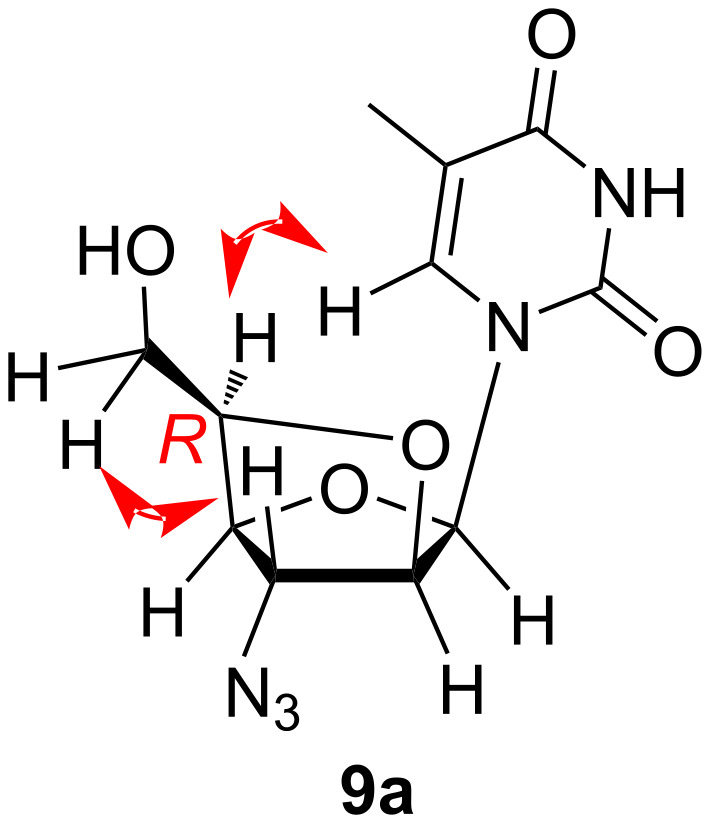
Structure elucidation of (5′*R*)-3′-azido-3′-deoxy-2′-*O*,5′-*C*-bridged-β-ᴅ-homoarabinofuranosylthymine (**9a**).

The stereochemistry at the C5′ carbon in bicyclic homonucleosides **9a**,**b** was established on the basis of ^1^H,^1^H-COSY and NOESY NMR spectral data analysis of nucleoside **9a** (Figure 2). Herein, cross peaks were observed in the NOESY spectrum of compound **9a** due to the spatial interaction of the C6′ methylene protons resonating at δ 3.41 with the C3′ proton resonating at δ 4.73 and the C5′ proton resonating at δ 4.15 with the C6 proton of the nucleobase resonating at δ 7.67 (see Supporting Information File 1). Based on this analysis of the spatial arrangement of the groups attached at the C5′ chiral centre, the stereochemistry of this carbon centre was assigned to be (*R*) for both of these nucleosides.

**Scheme 6:**
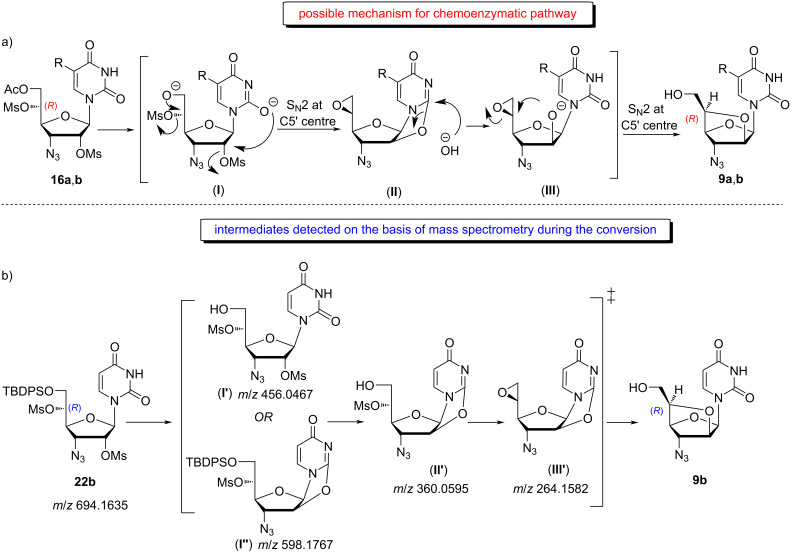
a) Plausible mechanism for the synthesis of (5′*R*)-3′-azido-3′-deoxy-2′-*O*,5′-*C*-bridged-β-ᴅ-homoarabinofuranosyl nucleosides **9a**,**b** from **16a**,**b** and b) intermediates detected on the basis of mass spectrometry during the conversion of **22b** into **9b**.

The study of NOESY NMR spectral data of compound **9a** enabled us to assign the stereochemistry at the C5′ chiral centre in the compound as (*R*). Following this trend, we assigned the same stereochemistry for the C5′ centre of nucleoside **9b**. Interestingly, we noticed that the stereochemistry of the C5′ centre was unaltered in the dimesylated allofuranosyl nucleosides **16a**,**b** or **22a**,**b** and the corresponding bridged homoarabinofuranosylpyrimidine nucleosides **9a**,**b**, whereas we expected an inversion of stereochemistry as it was noticed in earlier literature [30–31]. Depending on this observation, we postulated a mechanistic pathway for this cyclization process, where two consecutive inversions of configuration at the C5′ centre enabled the retention of stereochemistry. Thus, formation of 5,6-epoxide moieties was common in case of hexose carbohydrates, where the C6-OH group attacked the C5 centre and substituted the mesyl group present at C5 to form 5,6-epoxide moieties [32–34]. Herein, we elucidated the mechanistic pathway for conversion of **16a**,**b** into **9a**,**b** through the formation of an intermediate epoxide II, which has (*S*) stereochemistry at the C5′ due to first inversion of configuration by attack from the C6′-OH group (Scheme 6). The second inversion at the C5′ centre occurred when the C2′-OH attacked the same chiral centre and opened the epoxide ring by S_N_2 reaction and inverted the configuration of the centre into (*R*). In the chemical route for synthesis of bridged nucleosides **9a**,**b**, an unusual removal of TBDPS was observed when nucleosides **22a**,**b** were treated with 2 M NaOH in dioxane and water (1:1). A literature search showed that the TBDPS protecting group could be removed under basic conditions [35–36]. However, removal of the TBDPS group from the primary hydroxy group of a nucleoside under basic conditions of 2 M NaOH in dioxane and water (1:1) was the first of its kind in the literature. The progress of the reaction (from nucleoside **22b** to nucleoside **9b**) was investigated by mass spectrometry (MS) and a sample of the reaction mixture was collected during the reaction. The mass spectral analysis showed molecular ion peaks ([M + H]^+^) corresponding to the intermediates **I′**, **I′′**, **II′** and **III′** (Scheme 6), which illustrated the removal of TBDPS from the nucleoside and explained the retention of configuration at the C5′ centre. So, we assembled the identified intermediates in a most possible sequence to explain the progress of the reaction (Scheme 6). The sequence of the intermediates also described that epoxide formation occurred after removal of TBDPS and then attack from C2′-OH happened at the C5′ centre, i.e., two consecutive S_N_2 reactions at the C5′ centre, which retained the configuration of that chiral centre as (*R*) in the 2′-*O*,5′-*C*-bridged-β-ᴅ-homoarabinofuranosyl-nucleosides **9a**,**b**.

The structures of all the synthesized compounds **9a**,**b**, **11**, **12a**,**b**, **13a**,**b**, **14a**,**b**, **15a**,**b**, **16a**,**b**, **17**, **18**, **19**, **20a**,**b**, **21a**,**b**, and **22a**,**b** were unambiguously established on the basis of their spectral (IR, ^1^H, ^13^C NMR, ^1^H,^1^H-COSY NMR, ^1^H,^13^C-HETCOR NMR, NOESY NMR and HRMS) data analysis. The structures of known compounds **11** and **17** were further confirmed by comparison of their physical and spectral data with those reported in literature [28].

## Conclusion

The efficiency of Lipozyme^®^ TL IM was discovered for exclusive acetylation of the primary hydroxy group in 3′-azido-3′-deoxy-β-ᴅ-allofuranosylthymine and -uracil over the two secondary hydroxy groups present in the molecule. The enzymatically prepared monoacetylated 3′-azido-3′-deoxy-β-ᴅ-allofuranosylthymine and -uracil were conveniently converted into novel AZT bicyclic analogues, 3′-azido-3′-deoxy-2′-*O*,5′-*C*-bridged-homoarabinofuranosylthymine and -uracil in excellent yields. These nucleosides were also accessed through the chemical methodology in good yields. The biological activity, particularly antiviral activity study of these compounds will be carried out and reported separately.

## Supporting Information

File 1 Experimental part and NMR spectra.
