# Supplementary material for: Chemical and chemoenzymatic routes to bridged homoarabinofuranosylpyrimidines: Bicyclic AZT analogues
Source: Beilstein J Org Chem. 2022 Jan 11;18:95–101. doi: 10.3762/bjoc.18.10 (PMC8767562; doi:10.3762/bjoc.18.10)
Supplement: File 1 — Experimental part and NMR spectra. [file Beilstein_J_Org_Chem-18-95-s001.pdf]

BEILSTEIN JOURNAL OF ORGANIC CHEMISTRY

Supporting Information

for

Chemical and chemoenzymatic routes to bridged homoarabinofuranosylpyrimidines: Bicyclic AZT analogues

Sandeep Kumar, Jyotirmoy Maity, Banty Kumar, Sumit Kumar and Ashok K. Prasad

Beilstein J. Org. Chem. **2022,** *18,* 95–101. doi:10.3762/bjoc.18.10

Experimental part and NMR spectra

License and Terms: This is a supporting information file under the terms of the Creative Commons Attribution License (https://creativecommons.org/licenses/by/4.0). Please note that the reuse, redistribution and reproduction in particular requires that the author(s) and source are credited and that individual graphics may be subject to special legal provisions.
The license is subject to the *Beilstein Journal of Organic Chemistry* terms and conditions: (https://www.beilstein-journals.org/bjoc/terms)

Table of contents

Experimental S2

Synthesis of anomeric mixture of **12a**,**b** S2

General method for synthesis of **13a**,**b** S3–4

General method for synthesis of **14a**,**b** S5–6

General method for synthesis of **15a**,**b** S6–7

General method for synthesis of **16a**,**b** S7–8

General method for synthesis of **18** S8–9

General method for synthesis of **19a**,**b** S9–10

General method for synthesis of **20a**,**b** S10–11

General method for synthesis of **21a**,**b** S12–13

General method for synthesis of **22a**,**b** S13–14

General method for synthesis of **9a**,**b** S14–15

^{1}H and ^{13}C NMR spectra of compound **12a**,**b** S16

^{1}H and ^{13}C NMR spectra of compound **13a** S17

^{1}H and ^{13}C NMR spectra of compound **13b** S18

^{1}H and ^{13}C NMR spectra of compound **14a** S19

^{1}H and ^{13}C NMR spectra of compound **14b** S20

^{1}H and ^{13}C NMR spectra of compound **15a** S21

^{1}H and ^{13}C NMR spectra of compound **15b** S22

^{1}H and ^{13}C NMR spectra of compound **16a** S23

^{1}H and ^{13}C NMR spectra of compound **16b** S24

^{1}H and ^{13}C NMR spectra of compound **18** S25

^{1}H and ^{13}C NMR spectra of compound **19a**,**b** S26

^{1}H and ^{13}C NMR spectra of compound **20a** S27

^{1}H and ^{13}C NMR spectra of compound **20b** S28

^{1}H and ^{13}C NMR spectra of compound **21a** S29

^{1}H and ^{13}C NMR spectra of compound **21b** S30

^{1}H and ^{13}C NMR spectra of compound **22a** S31

^{1}H and ^{13}C NMR spectra of compound **22b** S32

^{1}H and ^{13}C NMR spectra of compound **9a** S33

DEPT-135 and COSY-NMR spectra of compound **9a** S34

HETCOR and NOESY spectra of compound **9a** S35
^{1}H and ^{13}C NMR spectra of compound **9b** S36
Mass spectrum of intermediates detected during the conversion of **22b** to **9b** S37

Experimental

All reagents and chemicals were purchased from Sigma-Aldrich Chemicals Pvt. Limited, India or from local commercial sources and were used without any further purification unless otherwise specified. R_f values of compounds are reported for analytical TLC using the specified solvents and 0.25 mm silica gel 60 F_{254} plates that were visualized by UV irradiation or by charring with 5% alcoholic sulfuric acid solution. Melting points were determined on a Büchi M-560 instrument and are uncorrected. Column chromatography was performed on silica gel (100–200 mesh). The IR spectra of compounds were recorded on a Perkin-Elmer model 2000 FT-IR spectrometer and are expressed as wavenumber (cm^{-1}). Specific rotation was measured on Rudolph Autopol II polarimeter. The ^{1}H, ^{13}C, ^{1}H-^{1}H COSY, HETCOR, NOESY and DEPT NMR spectra were recorded on Jeol alpha-400 spectrometer by using tetramethylsilane (TMS) as internal standard. The solvents were removed under reduced pressure using rotary evaporator, followed by further removal of the residual solvent under high vacuum. The chemical shift values are on δ scale and the coupling constant (*J*) are in Hz. HRMS analysis was carried out using Agilent G6530AA LC Q-TOF mass spectrometer using the ESI method.

Synthesis of 1,2,5,6-tetra-*O*-acetyl-3-azido-3-deoxy-α,β-D-allofuranose (12a,b)

To a solution of 1,2:5,6-di-*O*-isopropylidene-3-azido-3-deoxy-α-D-allofuranose (**11**, 3.0 g, 10.52 mmol) in acetic acid (60.12 mL, 1052 mmol) at 0 °C, acetic anhydride (9.94 ml, 105.2 mmol) and conc. sulfuric acid (0.056 mL, 1.05 mmol) were added. The reaction mixture was stirred for 3 h at room temperature and on completion, as indicated by TLC examination, quenched by adding cold water and neutralized with sodium bicarbonate. The compound was extracted with chloroform (3 × 80 mL) and the combined organic layer was washed with brine solution (2 × 80 mL) and dried over anhydrous sodium sulfate. The solvent was removed under reduced pressure and the crude product thus obtained was purified by column chromatography using ethyl acetate in petroleum ether as gradient solvent system to afford anomeric mixture **12a,b** (α:β = 1:3.5 based on integration of the anomeric proton in ^{1}H NMR).

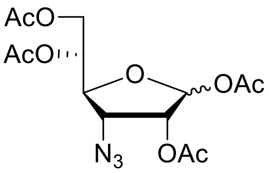

It was obtained as colourless viscous material oil (3.69 g) in 94% yield. R_f = 0.48 (20% ethyl acetate in petroleum ether); $[\alpha]_D^{24}$ = +138.02 (*c* 0.1, MeOH); IR (thin film) ν_{max}: 3024, 2112, 1744, 1434, 1371, 1212, 1098, 959, 755 and 668; ^{1}H NMR ($CDCl_3$, 400 MHz): δ 6.15 (1H, s), 5.34 (1H, d, *J* = 4.9 Hz), 5.15-5.17 (1H, m), 4.48 (1H, dd, *J* = 12.2, 3.2 Hz), 4.23 (1H, d, *J* = 7.2 Hz), 4.18 (1H, d, *J* = 4.8 Hz), 4.11 (1H, dd, *J* = 12.2, 5.6 Hz), 2.19 (3H, s), 2.15 (3H, s), 2.11 (3H, s) and 2.09 (3H, s); ^{13}C NMR ($CDCl_3$, 100.6 MHz): δ 170.6, 170.1, 169.5, 168.7, 98.1, 79.8, 75.8, 71.8, 62.3, 61.6, 21.0, 20.9, 20.8 and 20.6; HRMS (ESI): *m/z* cal. for $C_{14}H_{23}N_4O_9$ $[M+NH_4]^+$ = 391.1460; found: 391.1465.

General method for synthesis of 2′,5′,6′-tri-*O*-acetyl-3′-azido-3′-deoxy-β-D-allofuranosylpyrimidines 13a,b. To the anomeric mixture of tetra-*O*-acetylated sugar derivative (**12a,b**, 1.5 g, 4.02 mmol) and thymine/uracil (6.03 mmol) in anhydrous acetonitrile (60 mL), *N,O*-bis(trimethylsilyl)acetamide (3.93 mL, 16.08 mmol) was added dropwise. The reaction mixture was stirred at reflux for 1 h and then cooled to 0 °C. In the cooled reaction mixture trimethylsilyltriflouromethane sulfonate (1.23 mL, 6.83 mmol) was added slowly and the solution was refluxed for 3–4 h. On completion, the reaction was quenched with cold saturated aq. sodium bicarbonate solution (160 mL) and extracted with chloroform (3 × 120 mL). The combined organic layer was washed with saturated aq. sodium bicarbonate solution (2 × 120 mL), brine solution (2 × 120 mL) and then dried over anhydrous sodium sulfate to afford the crude product. The crude residue thus obtained was purified by silica gel column chromatography using ethyl acetate in petroleum ether as eluent to afford pure nucleosides **13a**/**13b** in good yields.

2′,5′,6′-Tri-*O*-acetyl-3′-azido-3′-deoxy-β-D-allofuranosylthymine (13a).

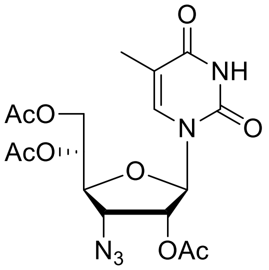

It was obtained as colourless viscous oil (1.62 g) in 92% yield. R_f= 0.45 (5 % MeOH in $CHCl_3$); $[\alpha]_D^{24}$ = +137.04 (*c* 0.1, MeOH); IR (KBr, cm^{-1}): 2110, 1739, 1687, 1462, 1371, 1211, 1043, 894, 785, 599 and 484cm^{-1}; ^1H NMR (400 MHz, $CDCl_3$): δ 9.45 (1H, s), 6.98 (1H, s), 5.63 (1H, d, *J* = 4.2 Hz), 5.51 (1H, dd, *J* = 6.4, 4.4 Hz), 5.33 (1H, dd, *J* = 9.0, 5.5 Hz), 4.53 (1H, d, *J* = 6.9 Hz), 4.48-4.50 (1H, m), 4.14 (1H, dd, *J* = 12.2, 5.4 Hz), 4.08 (1H, t, *J* = 6.3 Hz), 2.20 (3H, s), 2.16 (3H, s), 2.10 (3H, s) and 1.93 (3H, s); ^{13}C NMR (100 MHz, $CDCl_3$): δ 170.6, 170.1, 170.0, 163.8, 150.1, 137.0, 111.9, 90.8, 79.9, 74.7, 70.7, 62.1, 60.5, 20.9, 20.8, 20.5 and 12.5; HRMS (ESI): *m/z* calcd for $C_{17}H_{22}N_5O_9$ $[M+H]^+$ = 440.1412; found: 440.1420.

2′,5′,6′-Tri-*O*-acetyl-3′-azido-3′-deoxy-β-D-allofuranosyluracil (13b).

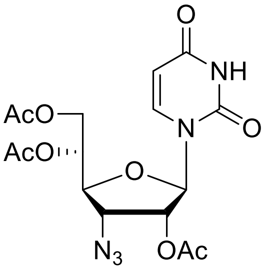

It was obtained as colourless viscous oil (1.59 g) in 93% yield. R_f = 0.50 (5% MeOH/chloroform); $[\alpha]_D^{24}$ = +122.58 (*c* 0.1, MeOH); IR (KBr, cm^{-1}): 3024, 2113, 1742, 1692, 1457, 1375, 1218, 1053, 814, 755 and 668; ^1H NMR (400 MHz, $CDCl_3$): δ 9.38 (1H, s), 7.17 (1H, d, *J* = 8.1 Hz),5.78 (1H, d, *J* = 8.1 Hz), 5.61 (1H, d, *J* = 4.1 Hz), 5.53 (1H, dd, *J* = 6.5, 4.1 Hz), 5.33 (1H, td, *J* = 5.6, 3.5 Hz), 4.52 (1H, d, *J* = 6.8 Hz), 4.49 (1H, dd, *J* = 9.5, 2.8 Hz), 4.14 (1H, dd, *J* = 12.3, 5.5 Hz), 4.09 (1H, t, *J* = 6.4 Hz), 2.20 (3H, s), 2.15 (3H, s) and 2.10 (3H, s); ^{13}C NMR (100 MHz, $CDCl_3$): δ 170.6, 170.1, 170.0, 163.0, 149.9, 141.4, 103.4, 91.4, 80.0, 74.8, 70.6, 62.1, 60.5, 20.9, 20.8 and 20.6**;** HRMS (ESI): *m/z* calcd for $C_{16}H_{20}N_5O_9$ $[M+H]^+$ = 426.1256; found: 426.1274.

General method of synthesis of 3′-azido-3′-deoxy-β-D-allofuranosylpyrimidines 14a, b. To a solution of compound **13a** (1.5 g, 3.41 mmol)/**13b** (1.5 g, 3.53 mmol) in methanol:water (9:1, 140 mL), K_2CO_3 (1.65 g, 11.94 mmol for **13a**)/(1.71 g, 12.36 mmol for **13b**) was added portion wise at 0 °C and the reaction mixture was stirred at 25 °C for 1 h. On completion of the reaction, solvent was removed under reduced pressure. The residue thus obtained was purified by column chromatography with a gradient solvent system of methanol in chloroform to afford trihydroxy nucleoside **14a**/**14b** in quantitative yields.

3′-Azido-3′-deoxy-β-D-allofuranosylthymine (14a).

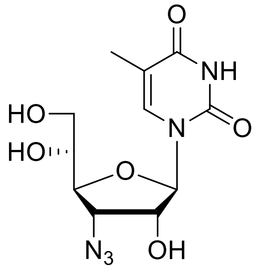

It was obtained as white solid (1.05 g) in 98% yield. R_f = 0.38 (10% MeOH/chloroform); $[\alpha]_D^{24}$ = +63.97 (*c* 0.1, MeOH); m/p: 167-170°C; IR (KBr, cm^{-1}): 2108, 1691, 1645, 1475, 1390, 1344, 1280, 1220, 1055, 1008, 918, 781, 607, 547 and 480; ^1H NMR (400 MHz, DMSO-d_6): δ 11.37 (1H, s), 7.68 (1H ,d, J = 1.1 Hz), 6.07 (1H, d, J = 5.4 Hz), 5.76 (1H, d, J = 6.6 Hz), 5.46 (1H, d, J = 4.7 Hz), 4.76 (1H, t, J = 5.0 Hz), 4.41 (1H, dd, J = 11.9, 5.8 Hz), 4.18 (1H, dd, J = 5.7, 3.1 Hz), 3.89 (1H, t, J = 3.3 Hz), 3.67-3.69 (1H, m), 3.41 (2H, t, J = 5.1 Hz) and 1.78 (3H, d, J = 0.9 Hz); ^{13}C NMR (100 MHz, DMSO-d_6): δ 163.7, 150.9, 136.0, 109.8, 86.4, 82.0, 73.7, 71.5, 62.4, 60.7 and 12.2; HRMS (ESI): *m/z* calcd for $C_{11}H_{16}N_5O_6$ $[M+H]^+$ = 314.1095; found: 314.1100.

3′-Azido-3′-deoxy-β-D-allofuranosyluracil (14b).

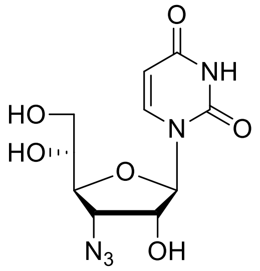

It was obtained as white solid (1.02 g) in 97% yield. R_f = 0.40 (10% MeOH/chloroform); $[\alpha]_D^{24}$ = +124.50 (*c* 0.1, MeOH); m/p:146-148°C; IR (KBr, cm^{-1}): 3392, 2114, 1693, 1468, 1421, 1392, 1262, 1108, 1070, 817, 718 and 649; ^1H NMR (400 MHz, DMSO-d_6): δ 11.37 (1H, s), 7.81 (1H,d, J = 8.1 Hz), 6.11 (1H, d, J = 5.3 Hz), 5.76 (1H, d, J = 6.5 Hz), 5.67 (1H, d, J = 8.1 Hz), 5.37 (1H, s), 4.74 (1H, s), 4.41 (1H, dd, J = 11.6, 5.8 Hz), 4.18 (1H, dd, J = 5.6, 3.2 Hz), 3.91 (1H, t, J = 3.3 Hz), 3.67 (1H, s) and 3.42 (2H, s); ^{13}C NMR (100 MHz, DMSO-d_6): δ 163.0, 150.8, 140.5, 102.2, 86.6, 82.2, 73.9, 71.4, 62.3 and 60.6; HRMS (ESI): *m/z* calcd for $C_{10}H_{14}N_5O_6$ $[M+H]^+$ = 300.0939; found: 300.0952.

General method for lipase-assisted synthesis of 6′-*O*-acetyl-3′-azido-3′-deoxy-β-D-allofuranosylpyrimidines 15a,b. To a solution of trihydroxy azidoallofuranosyl-pyrimidines

14a (1.0 g, 3.19 mmol)/**14b** (1.0 g, 3.34 mmol) in 2-Me-THF (60 mL) was added vinyl acetate (0.35 mL, 3.83 mmol for **14a**)/(0.37 mL, 4.01 mmol for **14b**) followed by addition of Lipozyme® TL IM (0.1 g, 10% w/w). The reaction mixture was stirred at 40 °C in an incubator shaker at 200 rpm for 2 h. On completion of the reaction as indicated by TLC examination, the reaction was quenched by filtering off the enzyme. The solvent was removed under reduced pressure and the residue thus obtained was purified by silica gel column chromatography using methanol in chloroform as gradient solvent system to afford the monoacetylated nucleosides **15a**/**15b** in quantitative yields.

6′-*O*-Acetyl-3′-azido-3′-deoxy-β-D-allofuranosylthymine (15a).

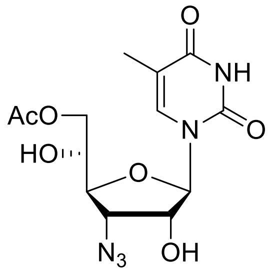

It was obtained as white solid (1.12g) in 98% yield. R_f = 0.45 (10% MeOH/chloroform); $[\alpha]_D^{24}$ = +93.76 (*c* 0.1, MeOH); m/p:155-158°C; IR (KBr, cm^{-1}): 3369, 2108, 1664, 1471, 1371, 1253, 1228, 1076, 1043, 908, 790, 594 and 549; ^1H NMR (400 MHz, DMSO-d_6): δ 11.37 (1H, s), 7.61 (1H, d, J = 1.1 Hz), 6.12 (1H, d, J = 5.4 Hz), 5.76 (2H, dd, J = 8.4, 4.5 Hz), 4.46 (1H, dd, J = 11.8, 5.8 Hz), 4.23 (1H, dd, J = 5.8, 3.3 Hz), 4.07 (1H, dd, J = 11.2, 4.4 Hz), 3.99 (1H, dd, J = 11.3, 6.3 Hz), 3.88-3.93 (1H, m), 3.80 (1H, dd, J = 4.7, 3.4 Hz), 2.03 (3H, s) and 1.79 (3H, d, J = 0.9 Hz); ^{13}C NMR (100 MHz, DMSO-d_6): δ 170.3, 163.6, 150.8, 136.1, 109.9, 86.8, 81.5, 73.1, 68.3, 64.9, 60.9, 20.7 and 12.1; HRMS (ESI): *m/z* calcd for $C_{13}H_{18}N_5O_7$ $[M+H]^+$ = 356.1201; found: 356.1213.

6′-*O*-Acetyl-3′-azido-3′-deoxy-β-D-allofuranosyluracil (15b).

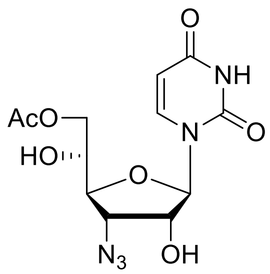

It was obtained as white solid (1.13 g) in 97% yield. R_f = 0.46 (10% MeOH/chloroform); $[\alpha]_D^{24}$ = +108.24 (*c* 0.1, MeOH); m/p: 128-130°C; IR (KBr, cm^{-1}):3394, 2113, 1682, 1466, 1385, 1260, 1082, 1049, 816, 770, 715 and 647; ^{1}H NMR (400 MHz, DMSO-d_6): δ 11.40 (1H, s), 7.77 (1H, d, *J* = 8.1 Hz), 6.18 (1H, d, *J* = 5.0 Hz), 5.75 (2H, d, *J* = 6.4 Hz), 5.67 (1H, d, *J* = 8.1 Hz), 4.45 (1H, dd, *J* = 11.0, 5.6 Hz), 4.22 (1H, dd, *J* = 5.5, 3.3 Hz), 4.05 (1H, dd, *J* = 11.3, 4.6 Hz), 3.99 (1H, dd, *J* = 11.3, 6.0 Hz), 3.90 (1H, s), 3.80-3.82 (1H, m) and 2.03 (3H, s); ^{13}C NMR (100 MHz, DMSO-d_6): δ 170.3, 163.0, 150.8, 140.7, 102.2, 87.1, 81.6, 73.3, 68.3, 64.8, 60.9 and 20.7; HRMS (ESI): *m/z* calcd for $C_{12}H_{16}N_5O_7$ $[M+H]^+$ = 342.1044; found: 342.1057.

General method for synthesis of 6′-*O*-acetyl-3′-azido-3′-deoxy-2′,5′-di-*O*-methanesulfonyl-*β*-D-allofuranosylpyrimidines 16a,b. To the stirred solution of **15a** (0.9 g, 2.53 mmol)/**15b** (0.9 g, 2.64 mmol) in anhydrous pyridine (10 mL), methanesulfonyl chloride (0.49 mL, 6.33 mmol for **15a**) and (0.51 mL, 6.60 mmol for **15b)** was added slowly at 0 °C and further stirred at 25 °C for 3-4 h. On completion of the reaction as indicated by TLC examination, the reaction mixture was poured over 10% ice cold hydrochloric acid solution (80 mL) to neutralize pyridine and the product was extracted with chloroform (3 × 80 mL). The combined organic layer was washed with saturated sodium bicarbonate solution (2 × 120 mL) and dried over anhydrous sodium sulfate. The solvent was removed under reduced pressure and the residue thus obtained was purified over silica gel column chromatography using methanol in chloroform as gradient to afford dimesylated nucleoside **16a**/**16b** in pure form.

6′-*O*-Acetyl-3′-azido-3′-deoxy-2′,5′-di-*O*-methanesulfonyl-β-D-allofuranosylthymine (16a).

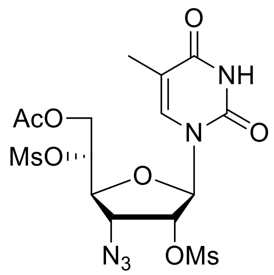

It was obtained as white solid (1.20 g) in 93% yield. R_f = 0.35 (5% MeOH/chloroform); $[\alpha]_D^{24}$ = +109.96 (*c* 0.1, MeOH); m/p:103-105°C; IR (KBr, cm^{-1}):2117, 1685, 1467, 1417, 1340, 1261, 1230, 1172, 1047, 964, 910, 835, 800, 677, 590, 522 and 484; ^{1}H NMR (400 MHz, $CDCl_3$): δ9.77 (1H, s), 7.23 (1H, s), 5.72 (1H, d, *J* = 3.3 Hz), 5.54-5.56 (1H, m), 5.17 (1H, s), 4.69 (1H, t, *J* = 6.4 Hz), 4.55 (1H, dd, *J* = 12.6, 2.7 Hz), 4.22 (1H, dd, *J* = 12.7, 6.4 Hz), 4.13 (1H, dd, *J*

= 6.4, 4.6 Hz), 3.34 (3H, s), 3.17 (3H, s), 2.14 (3H, s) and 1.92 (3H, s); ^{13}C NMR (100 MHz, $CDCl_3$): δ 170.4, 163.9, 150.6, 137.5, 112.3, 91.5, 79.8, 78.6, 77.1, 62.1, 59.4, 39.0, 38.5, 20.8 and 12.2; HRMS (ESI): *m/z* calcd for $C_{15}H_{22}N_5O_{11}S_2$ $[M+H]^+$ = 512.0752; found: 512.0770.

6′-*O*-Acetyl-3′-azido-3′-deoxy-2′,5′-di-*O*-methanesulfonyl-β-D-allofuranosyluracil (16b).

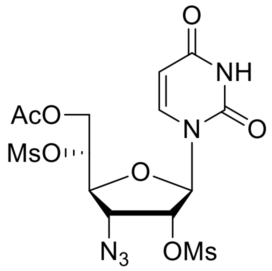

It was obtained as white solid (1.23 g) in 94% yield. R_f = 0.36 (5% MeOH/chloroform); $[\alpha]_D^{24}$ = +146.35 (*c* 0.1, MeOH); m/p: 133-135°C; IR (KBr, cm^{-1}): 2117, 1690, 1458, 1354, 1263, 1231, 1175, 1050, 916, 813 and 758; ^{1}H NMR (400 MHz, $CDCl_3$): δ 10.00 (1H, s), 7.42 (1H, d, *J* = 8.1 Hz), 5.79 (1H, d, *J* = 8.0 Hz), 5.73 (1H, d, *J* = 3.1 Hz), 5.58 (1H, dd, *J* = 6.1, 3.3 Hz), 5.16 (1H, t, *J* = 6.9 Hz), 4.68 (1H, t, *J* = 6.7 Hz), 4.54 (1H, dd, *J* = 12.6, 2.8 Hz), 4.23 (1H, dd, *J* = 12.7, 6.5 Hz), 4.15 (1H, dd, *J* = 7.1, 4.8 Hz), 3.25 (3H, s), 3.17 (3H, s) and 2.13 (3H, s); ^{13}C NMR (100 MHz, DMSO-d_6): δ 170.5, 163.5, 150.5, 142.2, 103.3, 92.1, 79.6, 78.9, 77.1, 62.1, 59.4, 38.9, 38.4 and 20.8; HRMS (ESI): *m/z* calcd for $C_{14}H_{20}N_5O_{11}S_2$ $[M+H]^+$ = 498.0595; found: 498.0611.

Synthesis of 6-*C*-(*tert*-butyldiphenylsilyloxymethyl)-1,2-*O*-isopropylidene-3-azido-3-deoxy-α-D-allofuranose (18)

To a solution of 1,2-*O*-isopropylidene-3-azido-3-deoxy-α-D-allofuranose (**17**, 6.0 g, 24.46 mmol) in pyridine (5.91 mL, 73.40 mmol) at 0° C, *tert*-butyl(chloro)diphenylsilane, i.e., TBDPS-Cl (6.91 ml, 26.91 mmol) and Tetra-n-butylammonium bromide (TBAB) (1.57 g, 4.89 mmol) were added. The reaction mixture was stirred for 12 h at room temperature and on completion, as indicated by TLC examination, quenched by adding cold water and neutralized with 10% hydrochloric acid solution. The compound was extracted with ethyl acetate (3 × 110 mL) and the combined organic layer was washed with brine solution (2 × 90 mL) and dried over anhydrous sodium sulfate. The solvent was removed under reduced pressure and the crude product thus obtained was purified by column chromatography using ethyl acetate in petroleum ether as gradient solvent system to afford **18** in pure form.

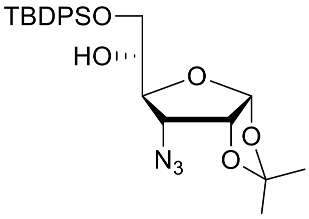

It was obtained as colourless viscous material (10.88 g) in 92% yield. R_f = 0.53 (30% ethyl acetate in petroleum ether); $[\alpha]_D^{24}$ = +25.36 (*c* 0.1, MeOH); IR (thin film) ν_{max}: 3040, 2117, 1695, 1313, 1118, 998, 959 and 750; ^{1}H NMR ($CDCl_3$, 400 MHz): δ 7.67 (4H, d, *J* = 6.8 Hz), 7.38-7.46 (6H, m), 5.77 (1H, d, *J* = 3.6 Hz), 4.72 (1H, t, *J* = 4.2 Hz), 4.18 (1H, dd, *J* = 9.2, 5.4 Hz), 3.86-3.91 (1H, m), 3.76-3.84 (2H, m), 3.59 (1H, dd, *J* = 9.2, 4.8 Hz), 2.62 (1H, d, *J* = 4.7 Hz), 1.56 (3H, s), 1.36 (3H, s) and 1.07 (9H, s); ^{13}C NMR ($CDCl_3$, 100.6 MHz): δ 135.6, 135.5, 132.9, 132.8, 130.0, 129.9, 127.9, 113.2, 104.0, 80.7, 77.2, 72.1, 64.5, 61.8, 26.9, 26.6, 26.5 and 19.3; HRMS (ESI): *m/z* cal. for $C_{25}H_{34}N_3O_5Si$ $[M+H]^+$ = 484.2262; found: 484.2268.

Synthesis of 6-*C*-(*tert*-butyldiphenylsilyloxymethyl)-1,2,5-tri-*O*-acetyl-3-azido-3-deoxy-*α,β*-D-allofuranose (19a,b)

To a solution of 6-*C*-(*tert*-Butyldiphenylsilyloxymethyl)-1,2-*O*-isopropylidene-3-azido-3-deoxy-α-D-allofuranose (**18**, 8.5 g, 17.57 mmol) in acetic acid (100.51 mL, 1757.54 mmol) at 0° C, acetic anhydride (16.61 ml, 175.75 mmol) and conc. sulfuric acid (0.09 mL, 1.76 mmol) were added. The reaction mixture was stirred for 4h at room temperature and on completion, as indicated by TLC examination, quenched by adding cold water and neutralized with sodium bicarbonate. The compound was extracted with ethyl acetate (3 × 120 mL) and the combined organic layer was washed with brine solution (2 × 100 mL) and dried over anhydrous sodium sulfate. The solvent was removed under reduced pressure and the crude product thus obtained was purified by column chromatography using ethyl acetate in petroleum ether as gradient solvent system to afford anomeric mixture **19a,b** (α:β = 1:5 based on integration of anomeric proton in ^{1}H NMR).

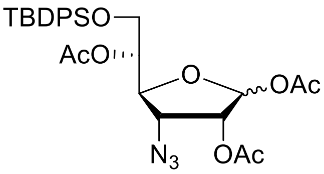

It was obtained as colourless viscous material oil (8.0 g) in 80% yield. R_f = 0.45 (30% ethyl acetate in petroleum ether); $[\alpha]_D^{24}$ = +46.01 (*c* 0.1, MeOH); IR (thin film) ν_{max}: 2940, 2115, 1745, 1311, 1112, 1098, 959 and 757; ^{1}H NMR ($CDCl_3$, 400 MHz): δ 7.64-7.67 (4H, m), 7.37-7.44

(6H, m), 6.14 (1H, s), 5.32 (1H, d, *J* = 5.0 Hz), 5.09 (1H, dd, *J* = 11.0, 4.7 Hz), 4.39 (1H, t, *J* = 6.8 Hz), 4.19 (1H, dd, *J* = 7.1, 5.1 Hz), 3.84 (2H, d, *J* = 4.6 Hz), 2.17 (3H, s), 2.08 (3H, s), 2.01 (3H, s) and 1.06 (9H, s); ^{13}C NMR ($CDCl_3$, 100.6 MHz): δ 170.1, 169.6, 168.8, 135.6, 135.5, 133.0, 132.9, 130.0, 129.9, 127.9, 127.8, 98.2, 80.1, 76.2, 74.3, 62.4, 61.1, 26.8, 26.7, 21.0, 20.9, 20.5, and 19.3; HRMS (ESI): *m/z* cal. for $C_{28}H_{36}N_3O_8Si$ $[M+H]^+$ = 570.2266; found: 570.2270.

General method of synthesis of 6′-*C*-(*tert*-butyldiphenylsilyloxymethyl)-2′,5′-di-*O*-acetyl-3′-azido-3′-deoxy-β-D-allofuranosylpyrimidines (20a,b). To the anomeric mixture of tri-*O*-acetylated sugar derivative (**19a, b**, 2.5 g, 4.38 mmol) and thymine/uracil (6.58 mmol) in anhydrous acetonitrile (90 mL), *N*,*O*-bis(trimethylsilyl)acetamide (4.29 mL, 17.55 mmol) was added dropwise. The reaction mixture was stirred at reflux for 1 h and then cooled to 0 °C. In the cooled reaction mixture trimethylsilyltriflouromethane sulfonate (1.35 mL, 7.46 mmol) was added slowly and the solution was refluxed for 12–16 h. On completion, the reaction was quenched with cold saturated aq. sodium bicarbonate solution (180 mL) and extracted with chloroform (3 × 110 mL). The combined organic layer was washed with saturated aq. sodium bicarbonate solution (2 × 100 mL), brine solution (2 × 100 mL) and then dried over anhydrous sodium sulfate to afford the crude product. The crude residue thus obtained was purified by silica gel column chromatography using ethyl acetate in petroleum ether as eluent to afford pure nucleosides **20a**/**20b** in good yields.

6′-*C*-(*tert*-Butyldiphenylsilyloxymethyl)-2′,5′-di-*O*-acetyl-3′-azido-3′-deoxy-β-D-allofuranosylthymine (20a).

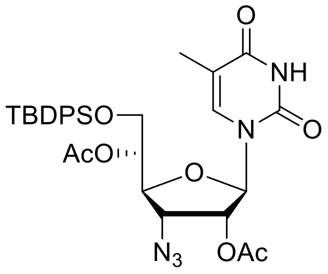

It was obtained as colourless viscous oil (2.51 g) in 90% yield. R_f= 0.45 (2% MeOH in $CHCl_3$); $[\alpha]_D^{24}$ = +68.05 (*c* 0.1, MeOH); IR (thin film) ν_{max}: 3240, 2195, 1740, 1210, 1095, 970 and 760; ^{1}H NMR (400 MHz, $CDCl_3$): δ 9.25 (1H, s), 7.64-7.66 (4H, m), 7.38-7.45 (6H, m), 6.91 (1H, s), 5.80 (1H, d, *J* = 5.2 Hz), 5.38 (1H, t, *J* = 5.9 Hz), 5.21 (1H, q, *J* = 4.7 Hz), 4.51 (1H, t, *J* = 6.1 Hz), 4.24 (1H, t, *J* = 5.1 Hz), 3.84 (2H, d, *J* = 4.6 Hz), 2.19 (3H, s), 2.10 (3H, s), 1.90 (3H,

s) and 1.07 (9H, s); ^{13}C NMR (100 MHz, $CDCl_3$): δ 170.1, 170.0, 163.6, 150.1, 135.8, 135.6, 135.5, 132.7, 130.0, 130.0, 127.9, 111.9, 88.4, 80.3, 74.7, 73.2, 62.2, 60.0, 26.8, 21.0, 20.5, 19.3 and 12.6; HRMS (ESI): *m/z* calcd for $C_{31}H_{38}N_5O_8Si$ $[M+H]^+$ = 636.2484; found: 636.2491.

6′-*C*-(*tert*-Butyldiphenylsilyloxymethyl)-2′,5′-di-*O*-acetyl-3′-azido-3′-deoxy-β-D-allofuranosyluracil (20b).

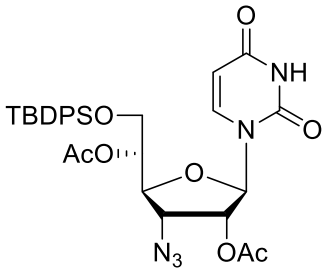

It was obtained as colourless viscous oil (2.51 g) in 92% yield. R_f = 0.46 (2% MeOH/chloroform); $[\alpha]_D^{24}$ = +68.05 (*c* 0.1, MeOH); IR (thin film) ν_{max}: 3145, 2190, 1742, 1205, 1120, 965 and 790; ^{1}H NMR (400 MHz, $CDCl_3$): δ 9.42 (1H, s), 7.64-7.66 (4H, m), 7.38-7.45 (6H, m), 7.11 (1H, d, *J* = 8.1 Hz), 5.78 (1H, d, *J* = 4.9 Hz), 5.73 (1H, d, *J* = 8.1 Hz), 5.37-5.40 (1H, m), 5.20 (1H, q, *J* = 4.7 Hz), 4.48 (1H, t, *J* = 6.1 Hz), 4.24 (1H, t, *J* = 5.3 Hz), 3.84 (2H, d, *J* = 4.7 Hz), 2.19 (3H, s), 2.09 (3H, s) and 1.07 (9H, s); ^{13}C NMR (100 MHz, $CDCl_3$): δ 170.1, 170.0, 163.0, 150.0, 140.2, 135.6, 135.5, 132.7, 132.6, 130.1, 130.0, 127.9, 103.4, 88.9, 80.3, 74.8, 73.1, 62.2, 60.0, 26.8, 21.0, 20.5 and 19.2; HRMS (ESI): *m/z* calcd for $C_{30}H_{36}N_5O_8Si$ $[M+H]^+$ = 622.2328; found: 622.2336.

General method of synthesis of 6′-*C*-(*tert*-butyldiphenylsilyloxymethyl)-3′-azido-3′-deoxy-β-D-allofuranosylpyrimidines (21a,b). To a solution of compound **20a** (1.9 g, 2.98 mmol)/**20b** (1.9 g, 3.05 mmol) in methanol/water (7:3, 120 mL), K_2CO_3 (0.82 g, 5.96 mmol for **20a**)/(0.84 g, 6.1 mmol for **20b**) was added portion wise at 0 °C and the reaction mixture was stirred at 25 °C for 4–6 h. On completion of the reaction, solvent was removed under reduced pressure. The residue thus obtained was purified by column chromatography with a gradient solvent system of methanol in chloroform to afford trihydroxy nucleoside **21a**/**21b** in quantitative yields.

6′-*C*-(*tert*-Butyldiphenylsilyloxymethyl)-3′-azido-3′-deoxy-β-D-allofuranosylthymine (21a).

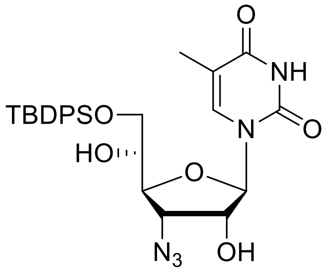

It was obtained as white solid (1.61 g) in 98% yield. R$_f$ = 0.46 (10% MeOH/chloroform); $[\alpha]_D^{24}$ = +67.12 (*c* 0.1, MeOH); m/p: 112-114°C; IR (KBr, cm^{-1}): 2912, 2135, 1680, 1642, 1320, 1260, 1205, 998, 870, 547 and 481; ^{1}H NMR (400 MHz, DMSO-d_6): δ 11.38 (1H, s), 7.64-7.67 (5H, m), 7.42-7.47 (6H, m), 6.12 (1H, d, *J* = 5.4 Hz), 5.82 (1H, d, *J* = 6.7 Hz), 5.63 (1H, d, *J* = 5.1 Hz), 4.47 (1H, dd, *J* = 12.0, 5.9 Hz), 4.28 (1H, dd, *J* = 5.5, 2.8 Hz), 4.07 (1H, s), 3.83-3.85 (1H, m), 3.68 (1H, d, *J* = 5.4 Hz), 3.17 (1H, d, *J* = 5.2 Hz), 1.78 (3H, s) and 1.00 (9H, s); ^{13}C NMR (100 MHz, DMSO-d_6): δ 163.7, 150.9, 136.0, 135.1, 135.0, 132.8, 129.9, 127.9, 109.8, 86.4, 82.1, 73.7, 70.8, 65.0, 60.6, 26.5, 18.8 and 12.2; HRMS (ESI): *m/z* calcd for $C_{27}H_{34}N_5O_6Si$ $[M+H]^+$ = 552.2273; found: 552.2280.

6′-*C*-(*tert*-Butyldiphenylsilyloxymethyl)-3′-azido-3′-deoxy-β-D-allofuranosyluracil (21b).

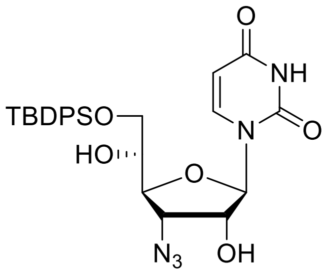

It was obtained as white solid (1.59 g) in 97% yield. R$_f$ = 0.47 (10% MeOH/chloroform); $[\alpha]_D^{24}$ = +105.22 (*c* 0.1, MeOH); m/p:123-125 °C; IR (KBr, cm^{-1}): 3092, 2120, 1690, 1460, 1405, 1301, 1222 1021, 815, 712 and 641; ^{1}H NMR (400 MHz, DMSO-d_6): δ 11.40 (1H, s), 7.79 (1H, d, *J* = 8.1 Hz), 7.63-7.67 (4H, m), 7.41-7.47 (6H, m), 6.15 (1H, d, *J* = 5.4 Hz), 5.81 (1H, d, *J* = 6.6 Hz), 5.67 (1H, dd, *J* = 8.1, 1.9 Hz), 5.57 (1H, d, *J* = 5.0 Hz), 4.46 (1H, dd, *J* = 11.8, 5.8 Hz), 4.27 (1H, dd, *J* = 5.5, 2.9 Hz), 4.08 (1H, t, *J* = 2.9 Hz), 3.81-3.83 (1H, m), 3.67 (1H, d, *J* = 5.5 Hz), 3.17 (1H, d, *J* = 3.7 Hz) and 1.00 (9H, s); ^{13}C NMR (100 MHz, DMSO-d_6): δ 162.9, 150.8, 140.5, 135.1, 135.0, 132.8, 129.9, 127.9, 102.2, 86.6, 82.1, 73.9, 70.8, 64.9, 60.6, 26.5 and 18.8; HRMS (ESI): *m/z* calcd for $C_{26}H_{32}N_5O_6Si$ $[M+H]^+$ = 538.2116; found: 538.2125.

General method for synthesis of 6′-*C*-(*tert*-butyldiphenylsilyloxymethyl)-3′-azido-3′-deoxy-2′,5′-di-*O*-methanesulfonyl-β-D-allofuranosylpyrimidines (22a,b). To the stirred solution of **21a** (1.3 g, 2.35 mmol)/**21b** (1.3 g, 2.42 mmol) in anhydrous pyridine (18 mL), methanesulfonyl chloride (0.402 mL, 5.17 mmol for **21a**) and (0.414 mL, 5.32 mmol for **21b**) was added slowly at 0 °C and further stirred at 25 °C for 8–10 h. On completion of the reaction as indicated by TLC examination, the reaction mixture was poured over 10% ice cold hydrochloric acid solution (90 mL) to neutralize pyridine and the product was extracted with chloroform (3 × 90 mL). The combined organic layer was washed with saturated sodium bicarbonate solution (2 × 110 mL) and dried over anhydrous sodium sulfate. The solvent was removed under reduced pressure and the residue thus obtained was purified over silica gel column chromatography using methanol in chloroform as gradient to afford dimesylated nucleoside **22a**/**22b** in pure form.

6′-*C*-(*tert*-Butyldiphenylsilyloxymethyl)-3′-azido-3′-deoxy-2′,5′-di-*O*-methanesulfonyl-β-D-allofuranosylthymine (22a).

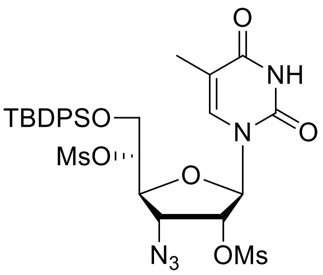

It was obtained as white solid (1.51 g) in 91% yield. R_f = 0.42 (5% MeOH/chloroform); $[\alpha]_D^{24}$ = +107.21 (*c* 0.1, MeOH); m/p: 90-92 °C; IR (KBr, cm^{-1}): 2992, 2117, 1680, 1521, 1317, 1303, 1271, 912, 830, 800 and 490; ^{1}H NMR (400 MHz, $CDCl_3$): δ 9.35 (1H, s), 7.64-7.68 (4H, m), 7.40-7.47 (6H, m), 7.33 (1H, s), 5.85 (1H, d, *J* = 4.7 Hz), 5.34-5.37 (1H, m), 5.02 (1H, dd, *J* = 8.3, 4.7 Hz), 4.57 (1H, t, *J* = 6.0 Hz), 4.17 (1H, dd, *J* = 5.7, 3.3 Hz), 3.86-3.94 (2H, m), 3.19 (3H, s), 3.01 (3H, s), 1.92 (3H, s) and 1.09 (9H, s); ^{13}C NMR (100 MHz, $CDCl_3$): δ 163.7, 150.5, 136.4, 135.6, 135.5, 132.2, 132.0, 130.4, 130.3, 128.1, 128.0, 112.4, 88.6, 80.2, 79.9, 78.1, 62.6, 58.9, 39.0, 38.5, 26.9, 19.2 and 12.2; HRMS (ESI): *m/z* calcd for $C_{29}H_{38}N_5O_{10}S_2Si$ $[M+H]^+$ = 708.1824; found: 708.1826.

6′-*C*-(*tert*-Butyldiphenylsilyloxymethyl)-3′-azido-3′-deoxy-2′,5′-di-*O*-methanesulfonyl-β-D-allofuranosyluracil (22b).

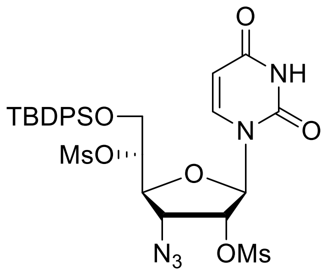

It was obtained as white solid (1.54 g) in 92% yield. R_f = 0.43 (5% MeOH/chloroform); $[\alpha]_D^{24}$ = +132.12 (*c* 0.1, MeOH); m/p: 102-103 °C; IR (KBr, cm^{-1}): 3011, 2116, 1692, 1457, 1260, 1110, 1032, 915, 812 and 757; ^{1}H NMR (400 MHz, $CDCl_3$): δ 9.62 (1H, s), 7.66 (4H, t, *J* = 7.5 Hz), 7.41-7.48 (7H, m), 5.78 (2H, dd, *J* = 11.2, 6.1 Hz), 5.39-5.42 (1H, m), 5.01 (1H, d, *J* = 4.0 Hz), 4.56 (1H, t, *J* = 6.1 Hz), 4.18-4.20 (1H, m), 3.89-3.92 (2H, m), 3.20 (3H, s), 3.01 (3H, s) and 1.08 (9H, s); ^{13}C NMR (100 MHz, $CDCl_3$): δ 163.1, 150.4, 141.1, 135.6, 135.5, 132.3, 132.1, 130.3, 130.2, 128.1, 128.0, 103.5, 89.5, 80.0, 79.7, 78.4, 62.6, 58.9, 38.9, 38.5, 26.9 and 19.2; HRMS (ESI): *m/z* calcd for $C_{28}H_{36}N_5O_{10}S_2Si$ $[M+H]^+$ = 694.1667; found: 694.1672.

General method for synthesis of (5′*R*)-3′-azido-3′-deoxy-2′-*O*,5′-*C*-bridged-β-D-homoarabinofuranosylpyrimidines (9a,b). To a solution of compound **16a/16b** and **22a/22b** (0.9 g, 1.76 mmol) in dioxane/water (1:1, 30 mL), 2 M NaOH (0.9 mL) was added at 0 °C and the reaction mixture was stirred for 12–24 h at 25 °C. On completion, acetic acid (10 mL) was added to neutralize the reaction followed by co-evaporated of the reaction solvent with toluene under reduced pressure. The residue thus obtained was purified by silica gel column chromatography using methanol in chloroform as gradient solvent system to afford nucleoside **9a/9b** in pure form.

(5′*R*)-3′-Azido-3′-deoxy-2′-*O*,5′-*C*-bridged-β-D-homoarabinofuranosylthymine (9a).

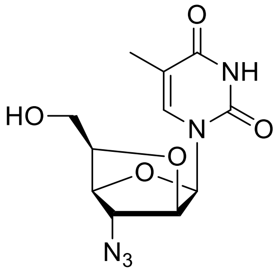

It was obtained as white solid (0.43 g) in 82% yield. R_f = 0.29 (5% MeOH/chloroform); $[\alpha]_D^{24}$ = +164.00 (*c* 0.1, MeOH); m/p: 178-180°C; IR (KBr, cm^{-1}): 3353, 2110, 1676, 1462, 1421, 1261,

1101, 1031, 962, 925, 844, 731, 599 and 489; ^{1}H NMR (400 MHz, DMSO-d_6): δ 11.40 (1H, s), 7.67 (1H, s), 5.80 (1H, s), 4.96 (1H, t, J = 5.6 Hz), 4.75 (1H, d, J = 2.3 Hz), 4.73 (1H, s), 4.59 (1H, d, J = 1.6 Hz), 4.15 (1H, t, J = 5.2 Hz), 3.41 (2H, t, J = 5.1 Hz) and 1.81 (3H, s); ^{13}C NMR (100 MHz, DMSO-d_6): δ 163.9, 150.4, 136.0, 108.3, 88.2, 83.9, 80.7, 75.4, 63.6, 61.0 and 12.2; HRMS (ESI): *m/z* calcd for $C_{11}H_{14}N_5O_5$ $[M+H]^+$ = 296.0989; found: 296.0998.

(5′*R*)-3′-Azido-3′-deoxy-2′-*O*,5′-*C*-bridged-β-D-homoarabinofuranosyluracil (9b).

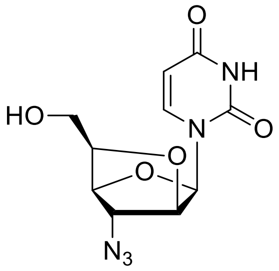

It was obtained as white solid (0.43 g) in 84% yield. R_f = 0.30 (5% MeOH/chloroform); $[\alpha]_D^{24}$ = +147.92 (*c* 0.1, MeOH); m/p: 163-165°C; IR (KBr, cm^{-1}): 3443, 2117, 1706, 1461, 1420, 1266, 1223, 1101, 1053, 911, 815, 754 and 664; ^{1}H NMR (400 MHz, DMSO-d_6):δ 11.41 (1H, s), 7.82 (1H, d, J = 8.1 Hz), 5.83 (1H, s), 5.59 (1H, d, J = 8.1 Hz), 4.97 (1H, t, J = 5.1 Hz), 4.75 (2H, d, J = 7.9 Hz), 4.60 (1H, s), 4.08 (1H, t, J = 5.0 Hz) and 3.40 (2H, s); ^{13}C NMR (100 MHz, DMSO-d_6): δ 163.2, 150.4, 140.7, 100.6, 88.3, 84.0, 80.7, 75.4, 63.7 and 61.0; HRMS (ESI): *m/z* calcd for $C_{10}H_{12}N_5O_5$ $[M+H]^+$ = 282.0833; found: 282.0842.

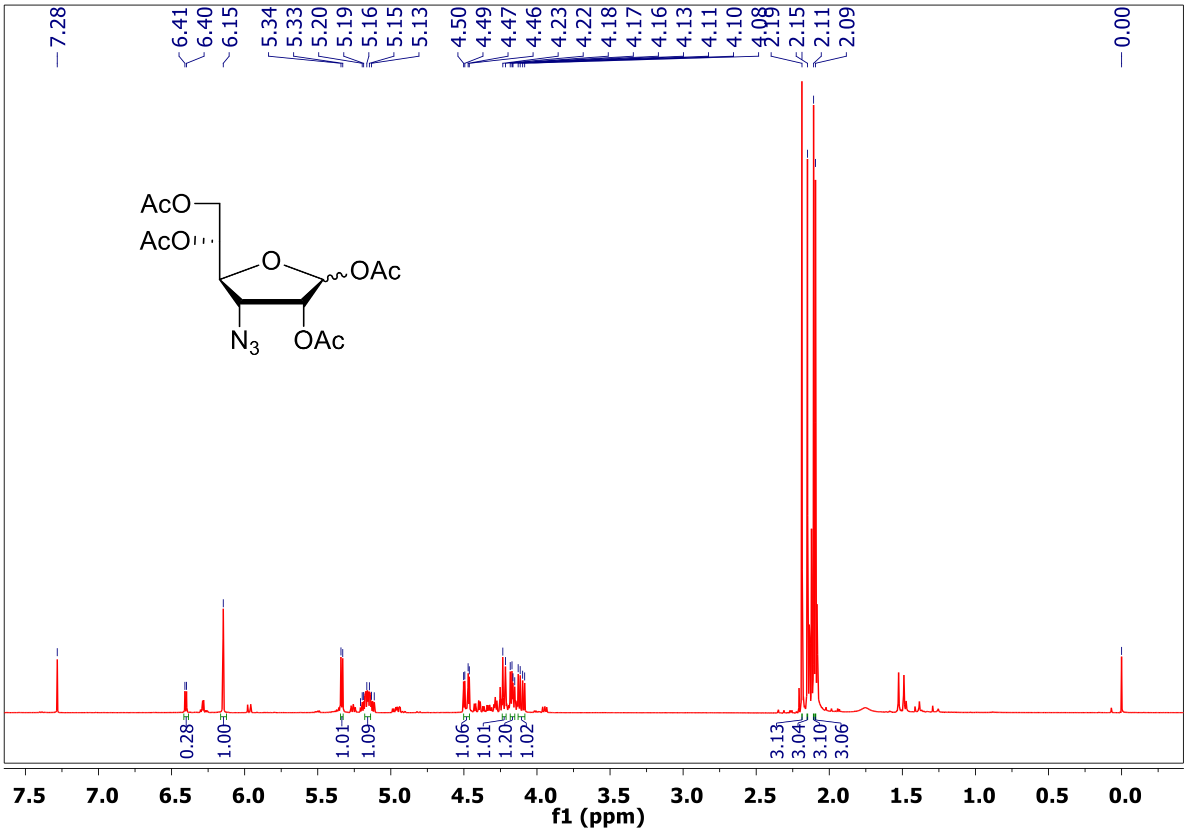

Figure S1: ^{1}H NMR spectrum of compound **12a, b** (400 MHz, $CDCl_3$).

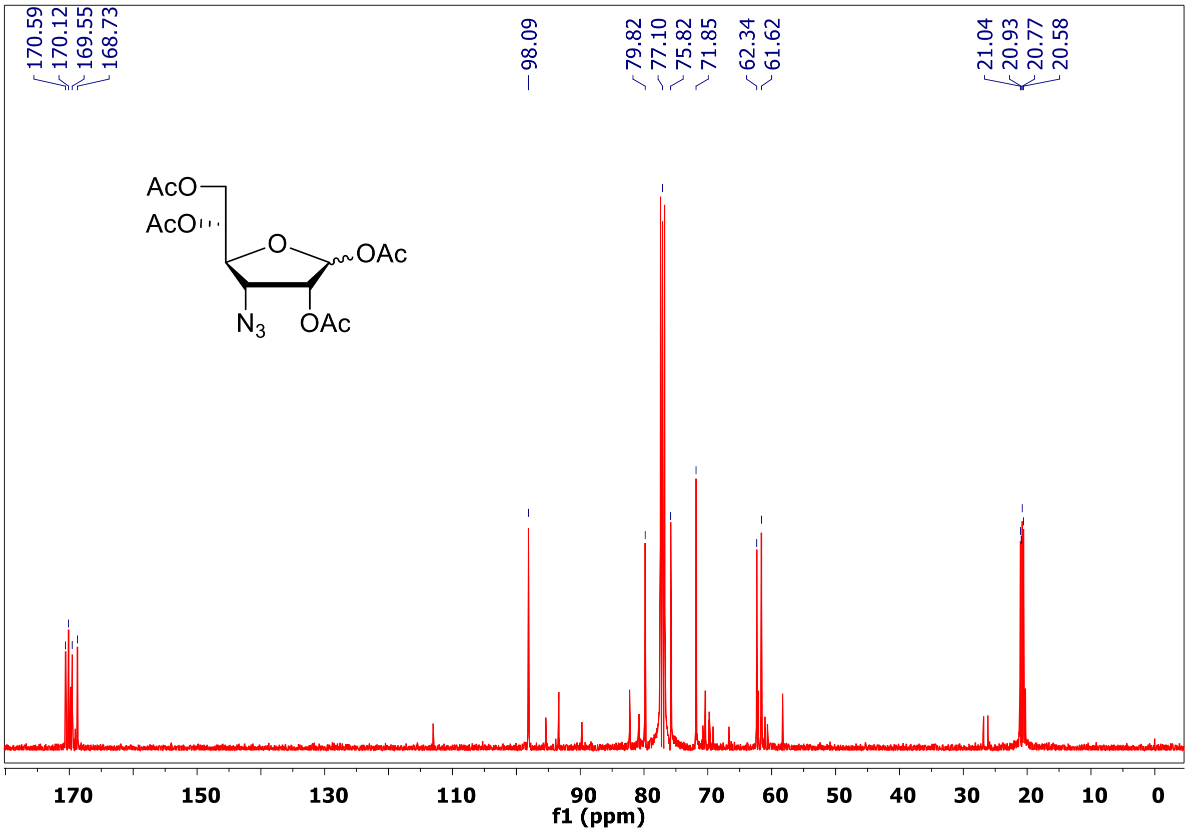

Figure S2: ^{13}C NMR spectrum of compound **12a, b** (100.6 MHz, $CDCl_3$).

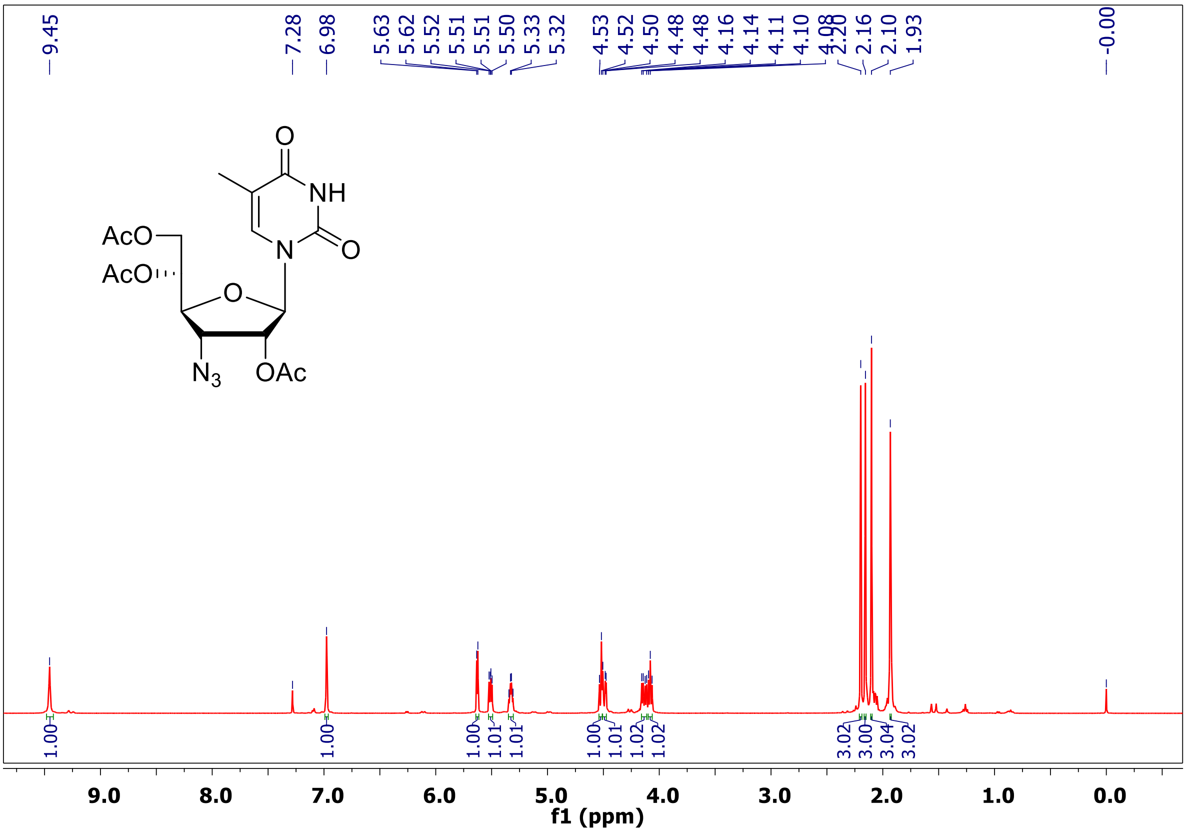

Figure S3: ^{1}H NMR spectrum of compound **13a** (400 MHz, $CDCl_3$).

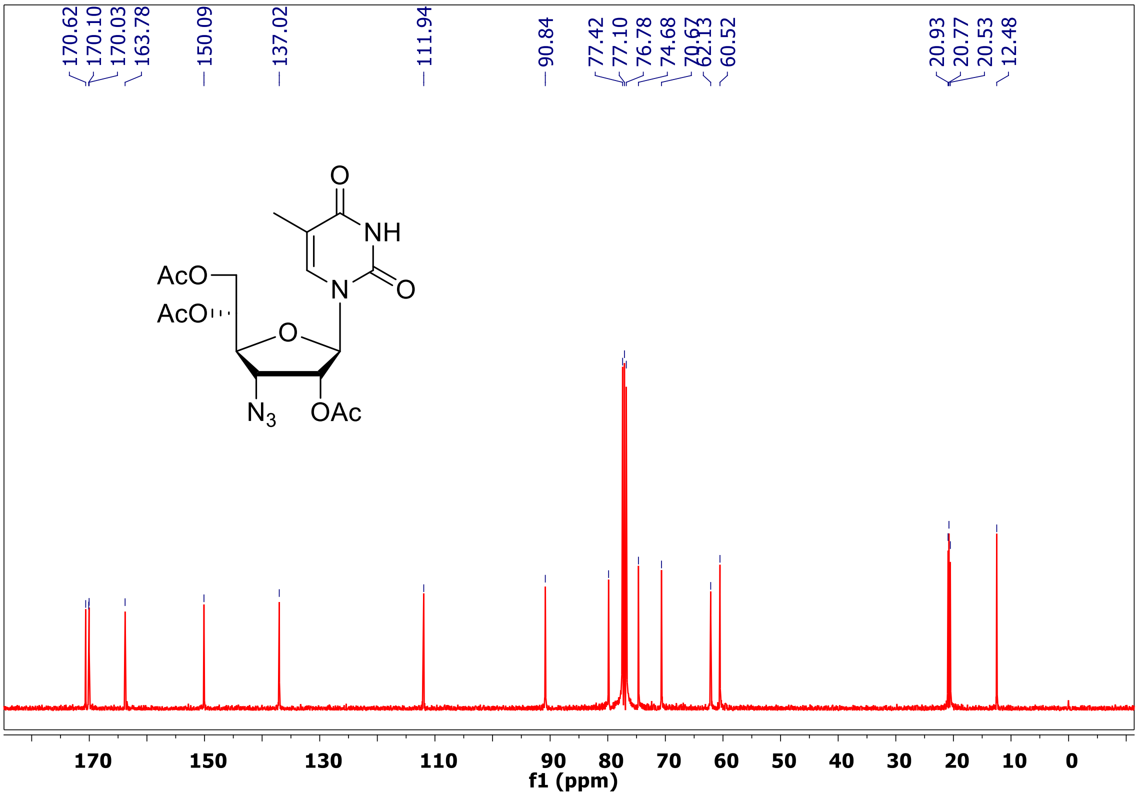

Figure S4: ^{13}C NMR spectrum of compound **13a** (100.6 MHz, $CDCl_3$).

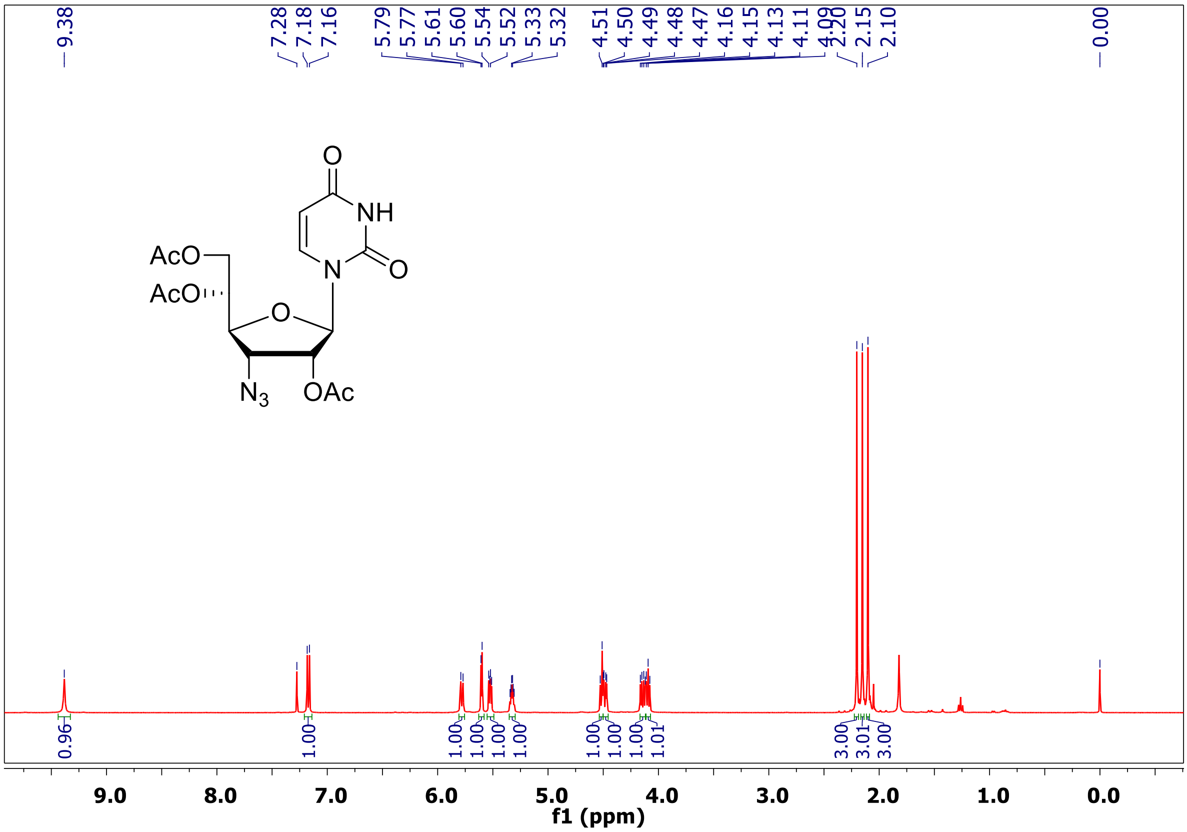

Figure S5: ^{1}H NMR spectrum of compound **13b** (400 MHz, $CDCl_3$).

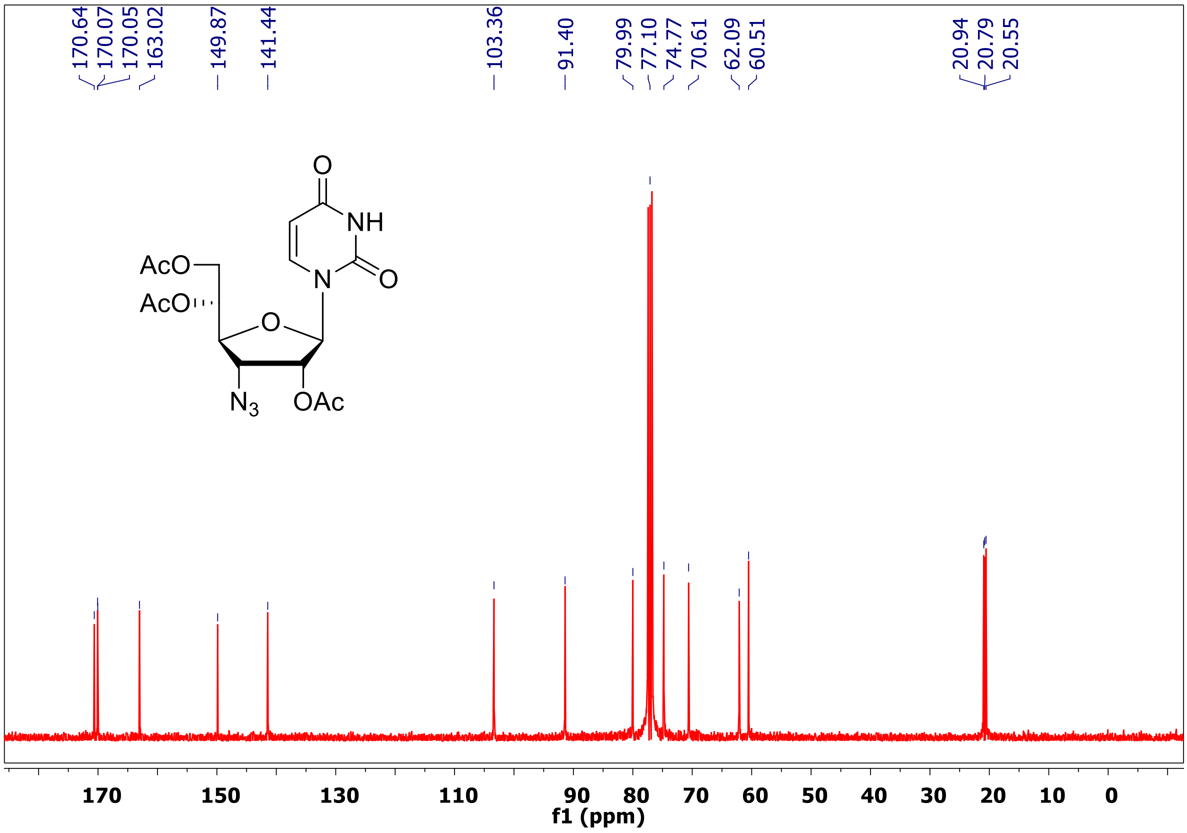

Figure S6: ^{13}C NMR spectrum of compound **13b** (100.6 MHz, $CDCl_3$).

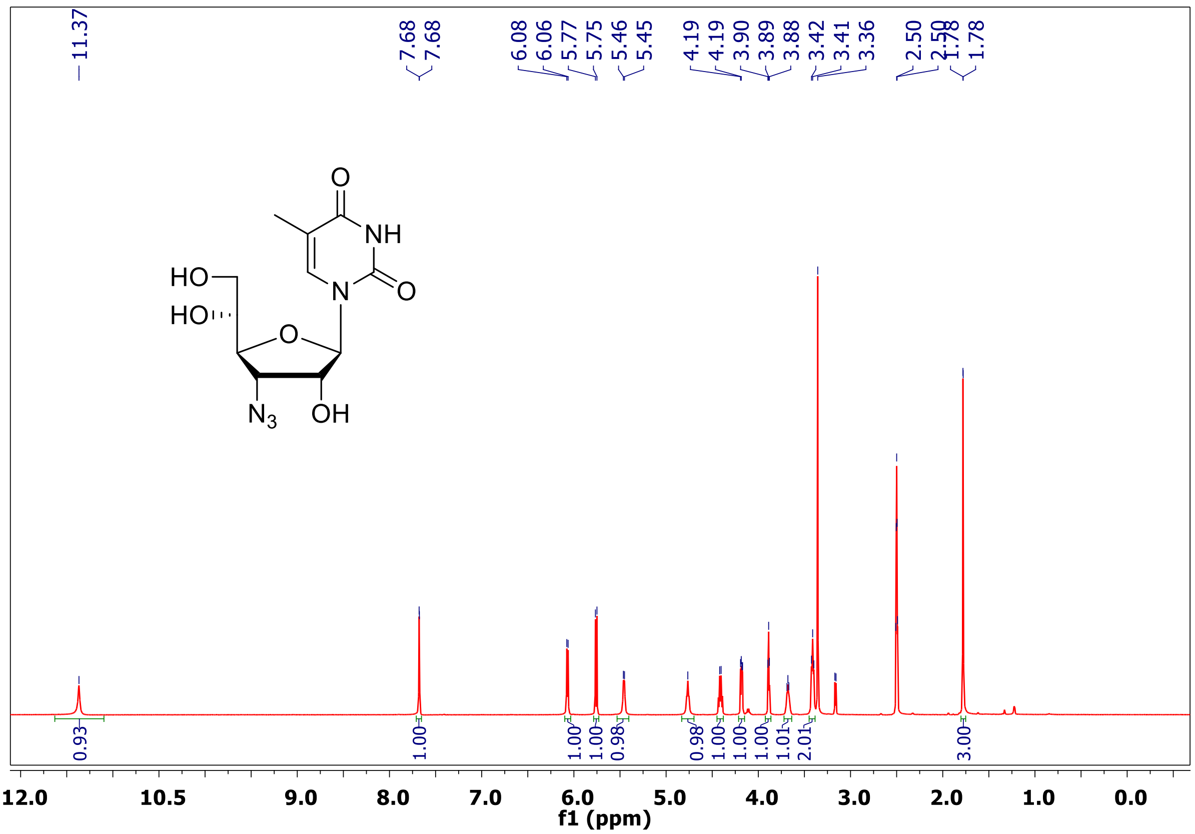

Figure S7: ^{1}H NMR spectrum of compound **14a** (400 MHz, DMSO-d_6).

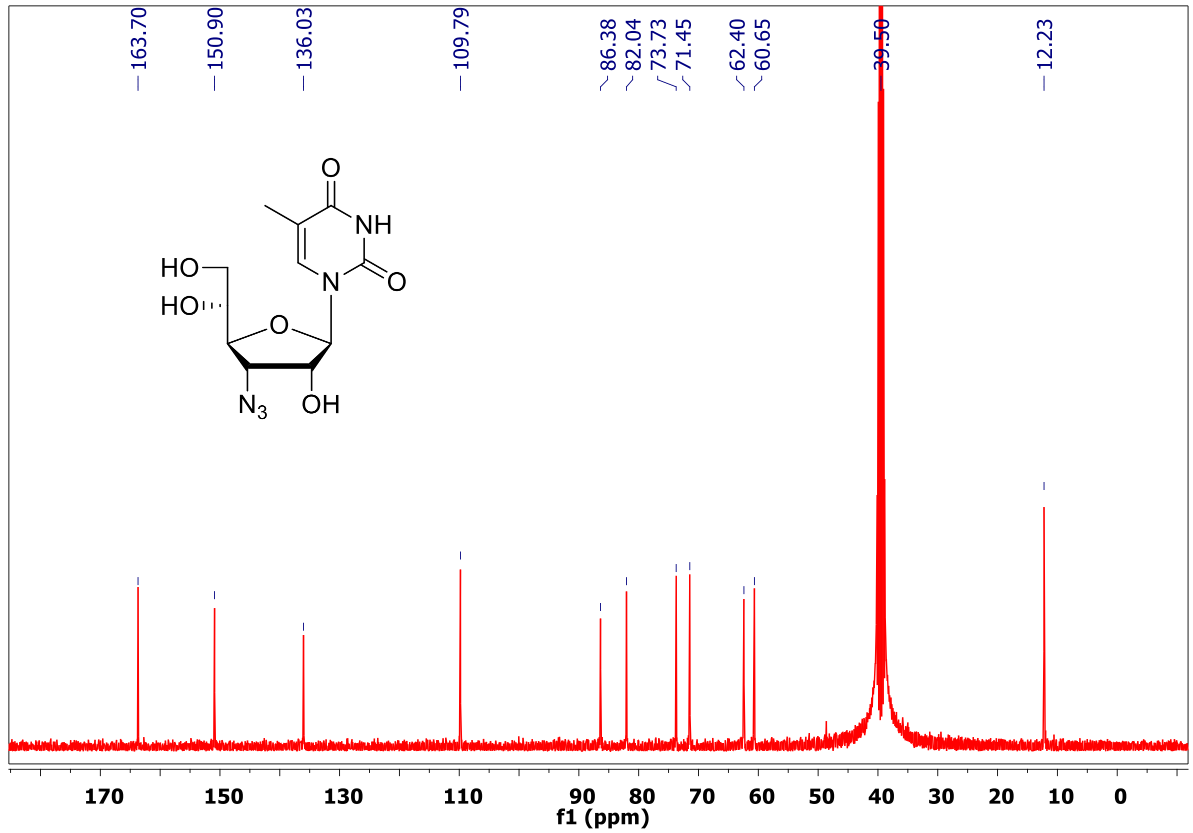

Figure S8: ^{13}C NMR spectrum of compound **14a** (100.6 MHz, DMSO-d_6).

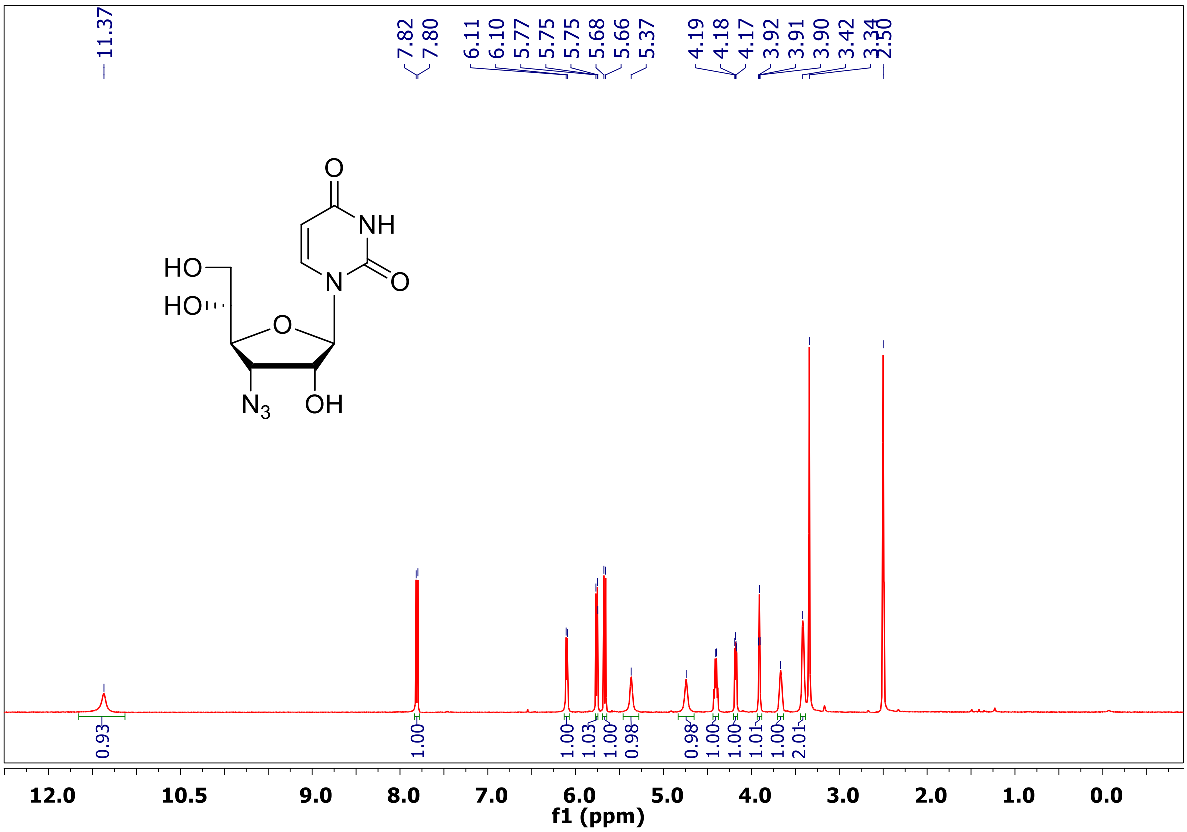

Figure S9: ^{1}H NMR spectrum of compound **14b** (400 MHz, DMSO-d_6).

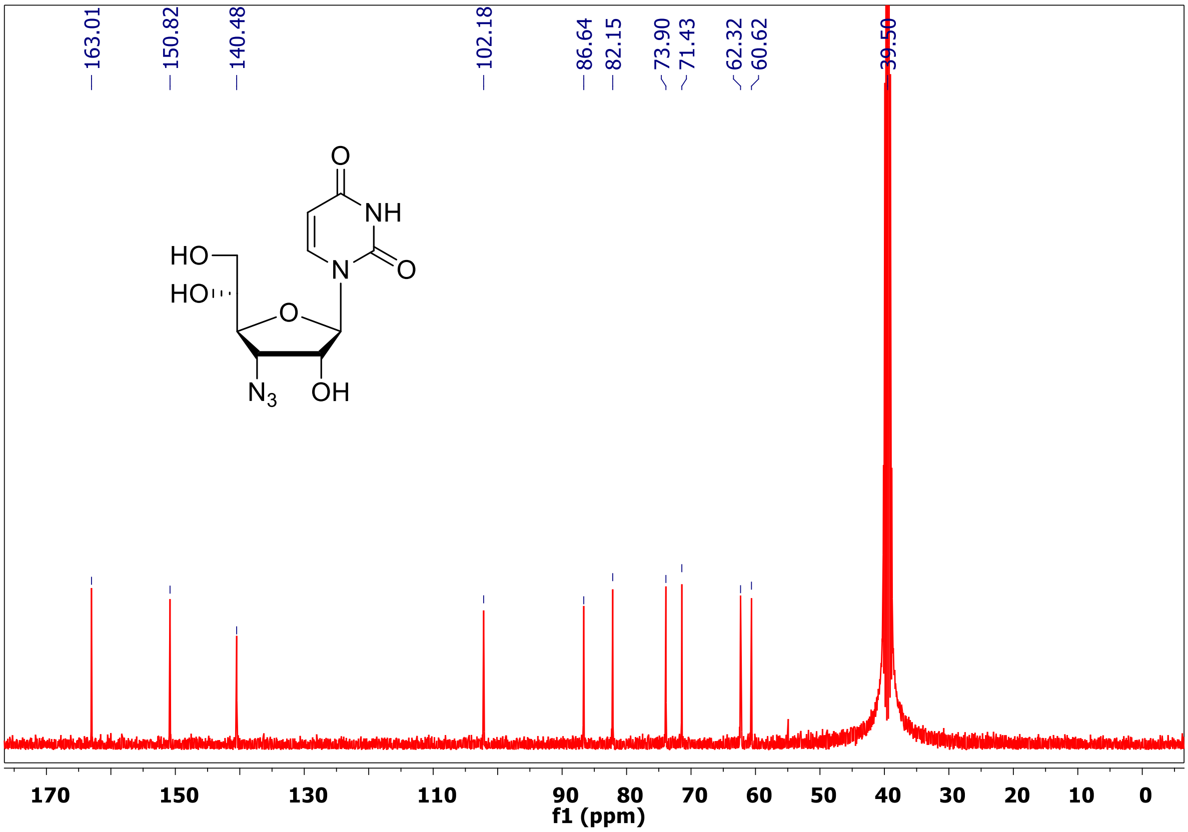

Figure S10: ^{13}C NMR spectrum of compound **14b** (100.6 MHz, DMSO-d_6).

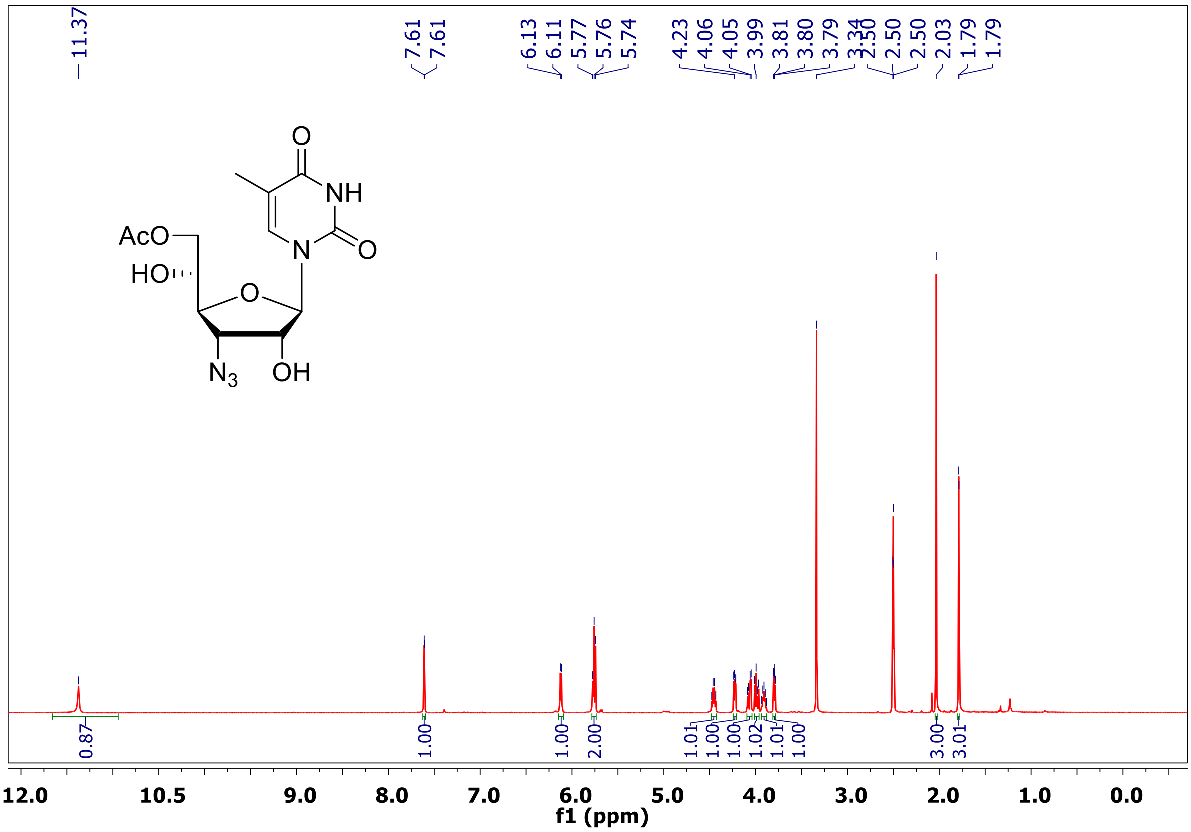

Figure S11: ^{1}H NMR spectrum of compound **15a** (400 MHz, DMSO-d_6).

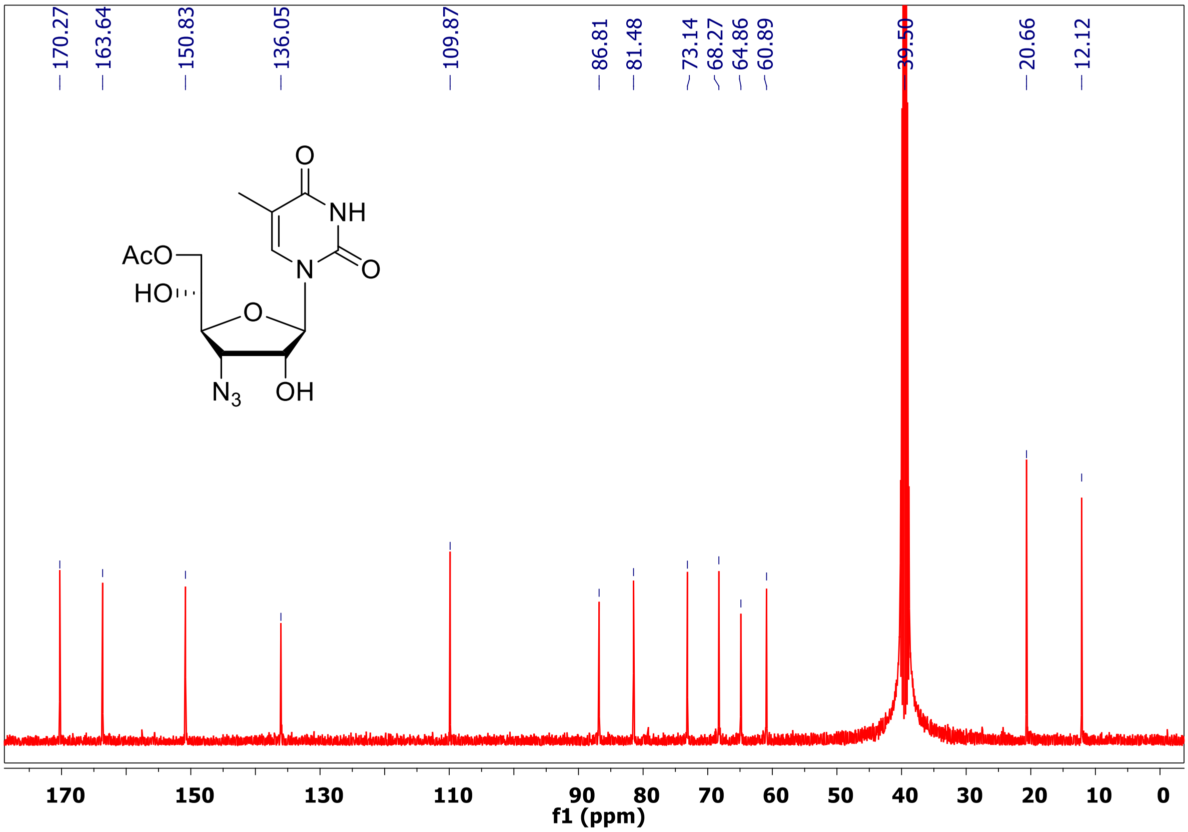

Figure S12: ^{13}C NMR spectrum of compound **15a** (100.6 MHz, DMSO-d_6).

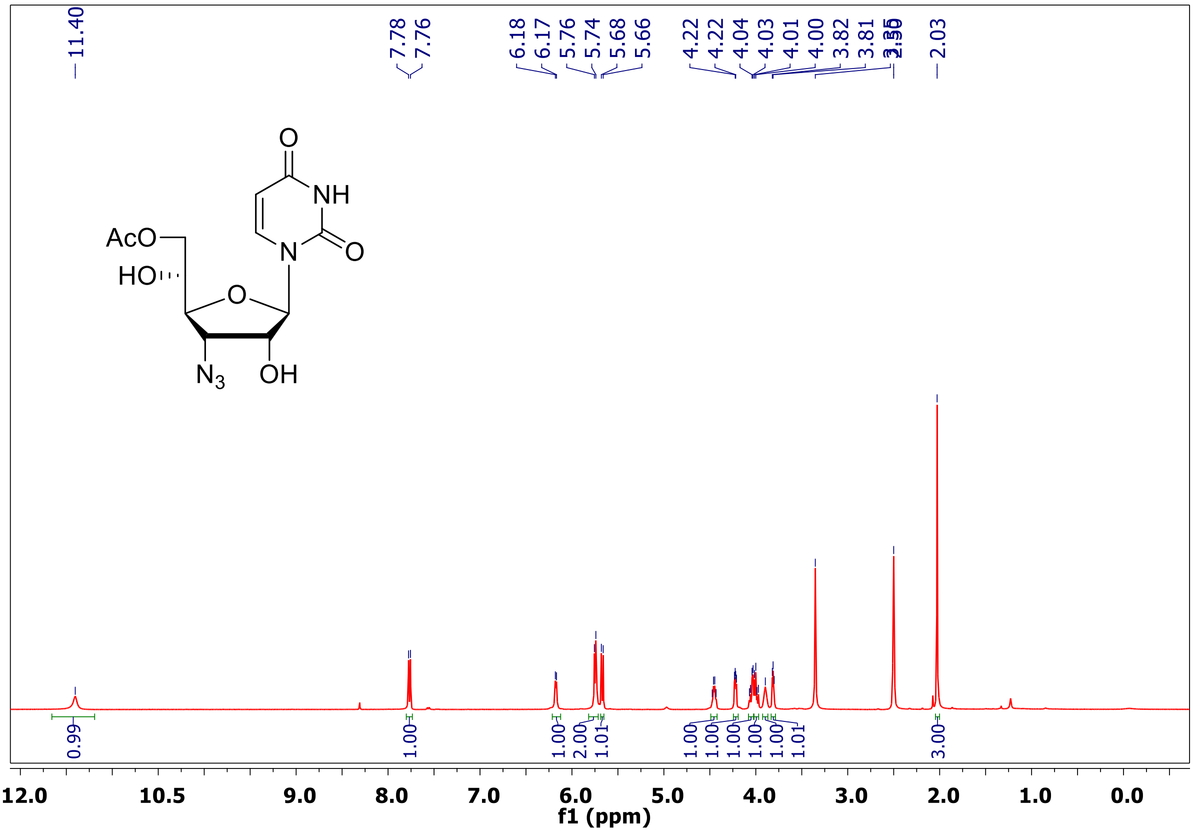

Figure S13: ^{1}H NMR spectrum of compound **15b** (400 MHz, DMSO-d_6).

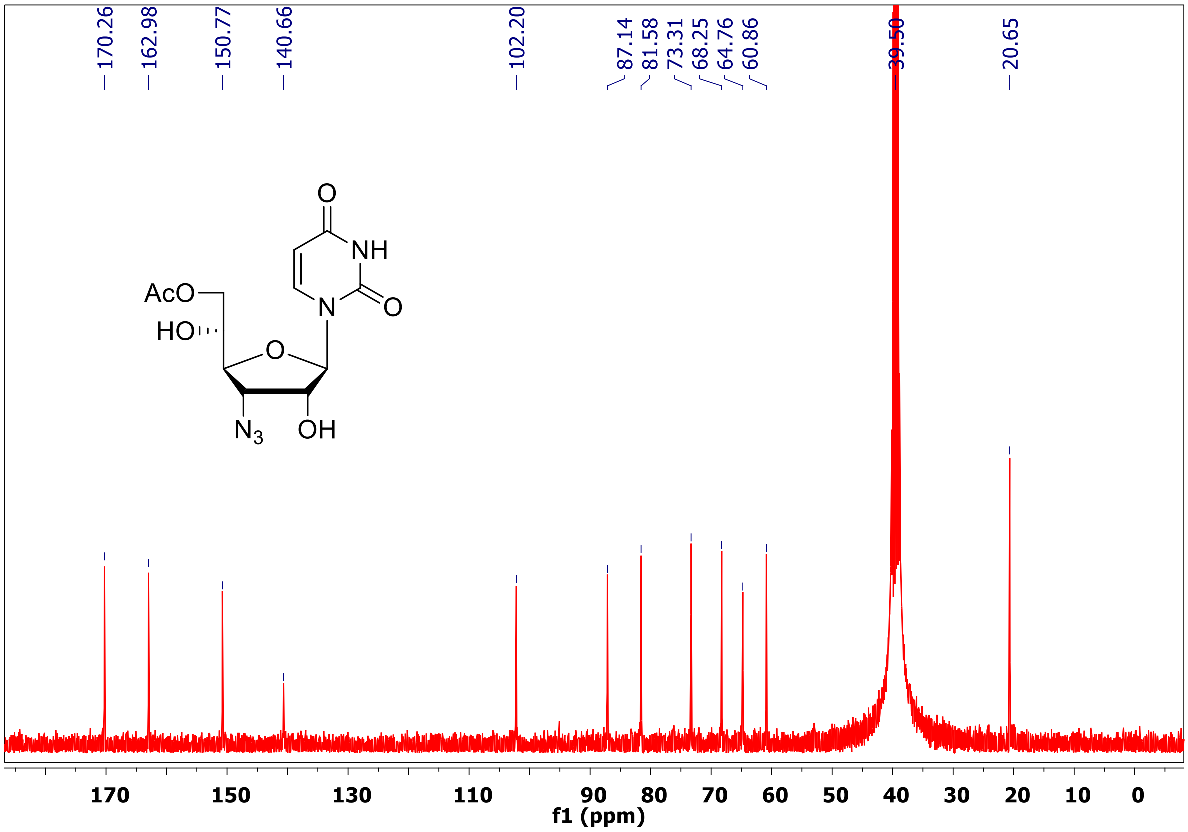

Figure S14: ^{13}C NMR spectrum of compound **15b** (100.6 MHz, DMSO-d_6)

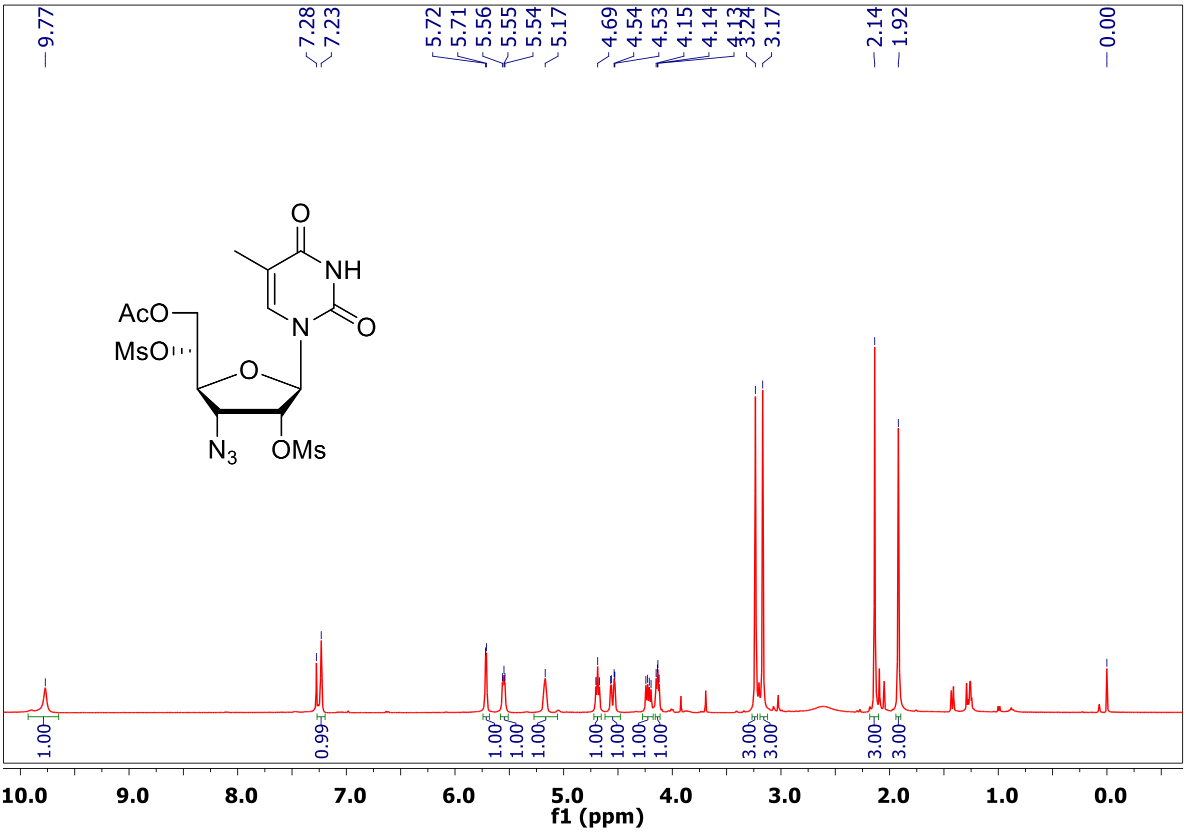

Figure S15: ^{1}H NMR spectrum of compound **16a** (400 MHz, $CDCl_3$).

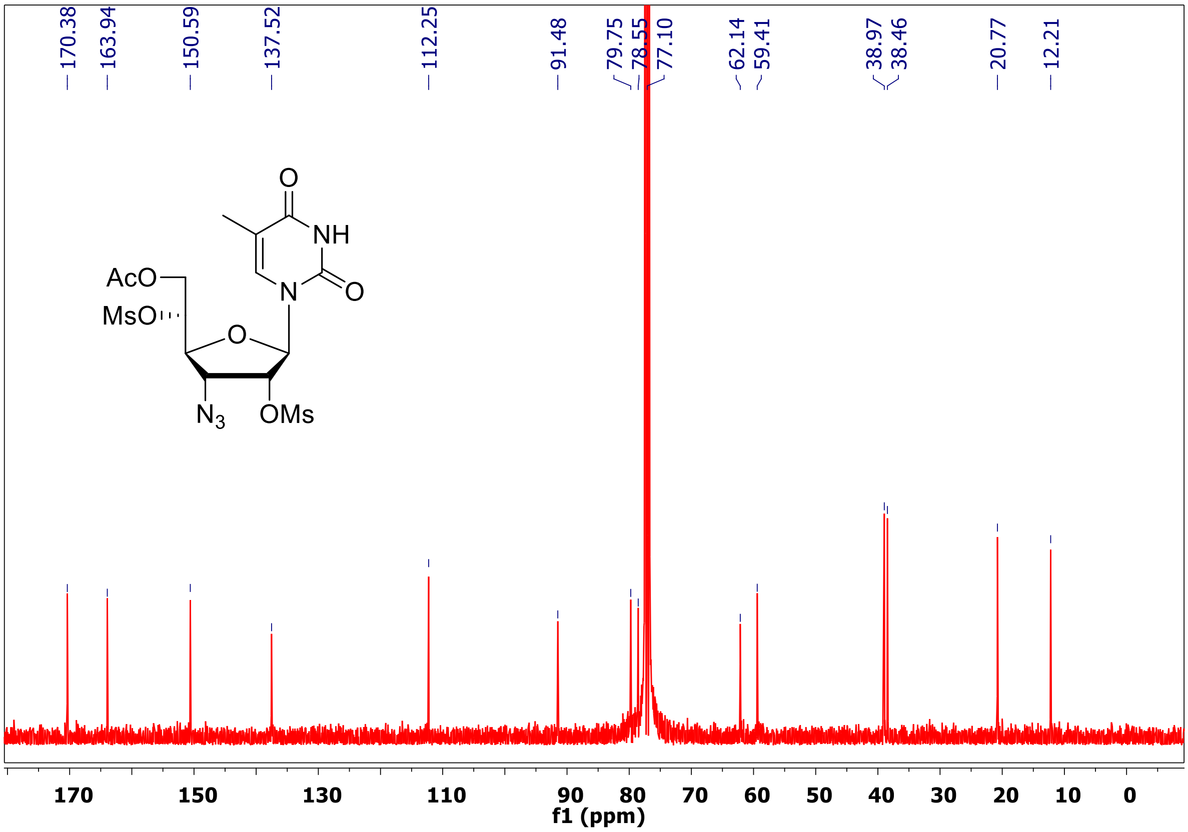

Figure S16: ^{13}C NMR spectrum of compound **16a** (100.6 MHz, $CDCl_3$).

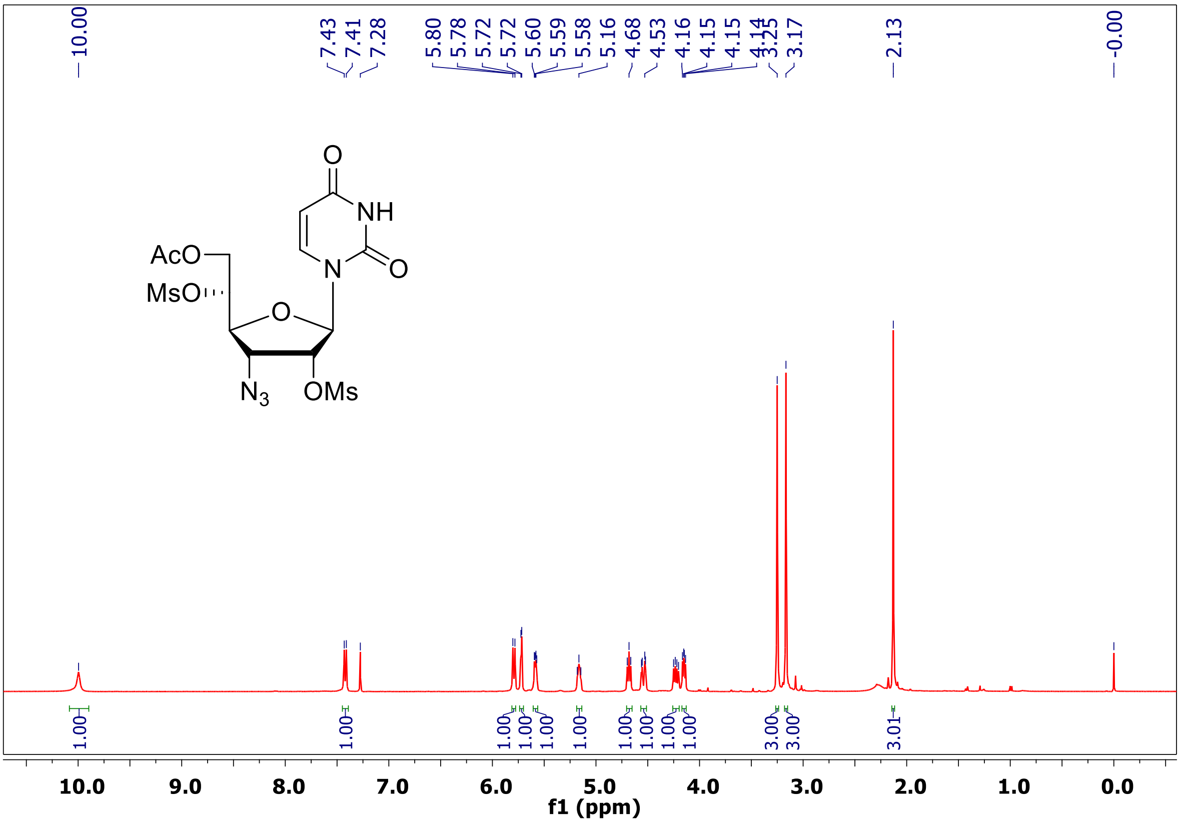

Figure S17: ^{1}H NMR spectrum of compound **16b** (400 MHz, $CDCl_3$).

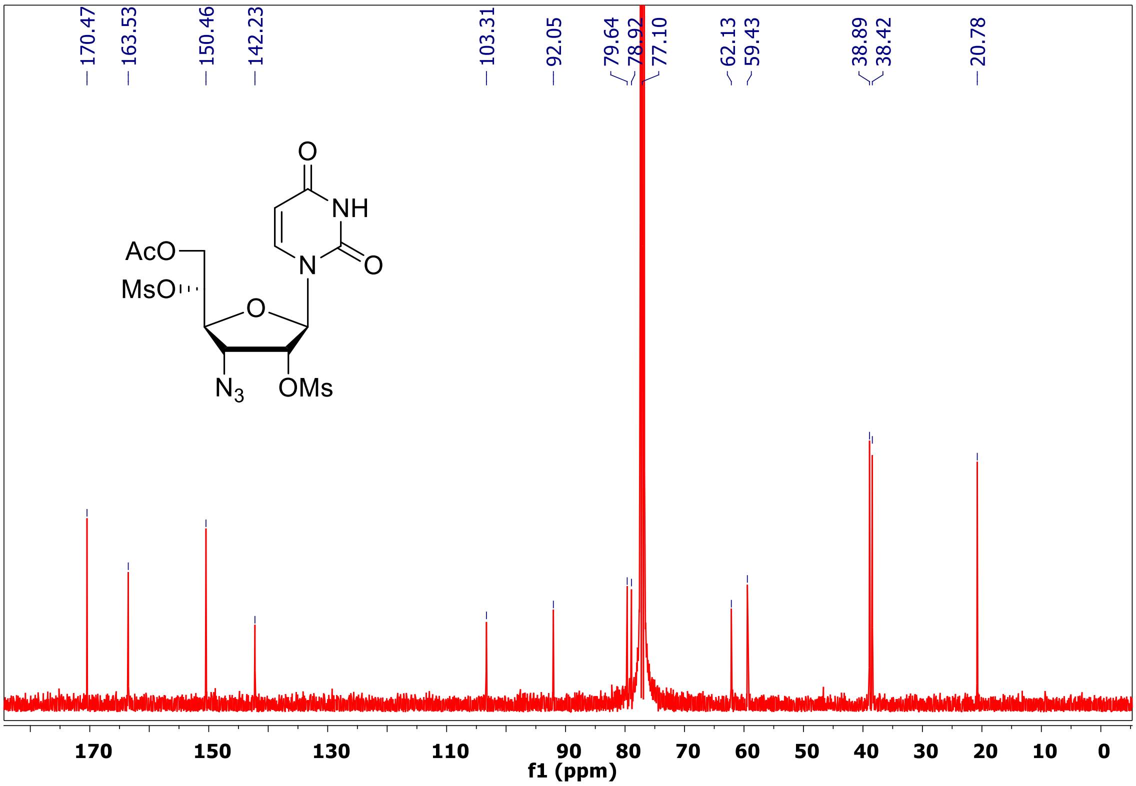

Figure S18: ^{13}C NMR spectrum of compound **16b** (100.6 MHz, $CDCl_3$).

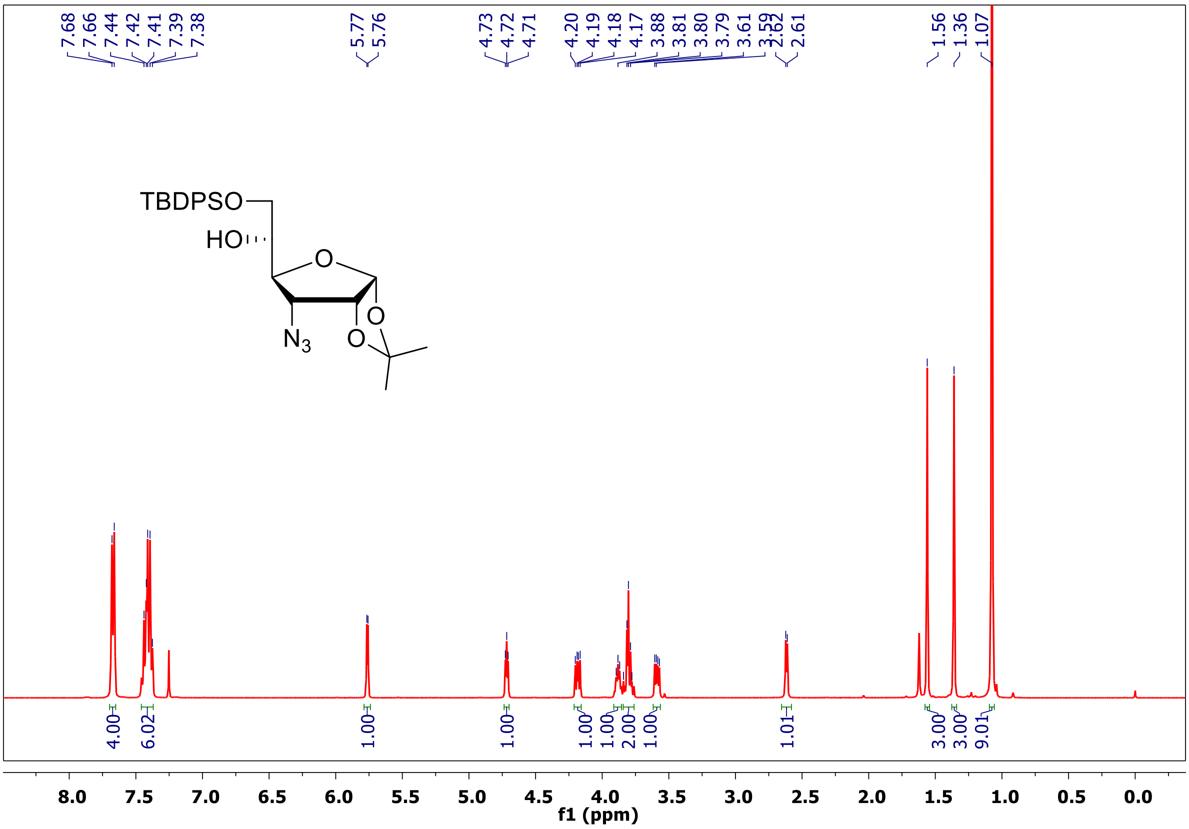

Figure S19: ^{1}H NMR spectrum of compound **18** (400 MHz, $CDCl_3$).

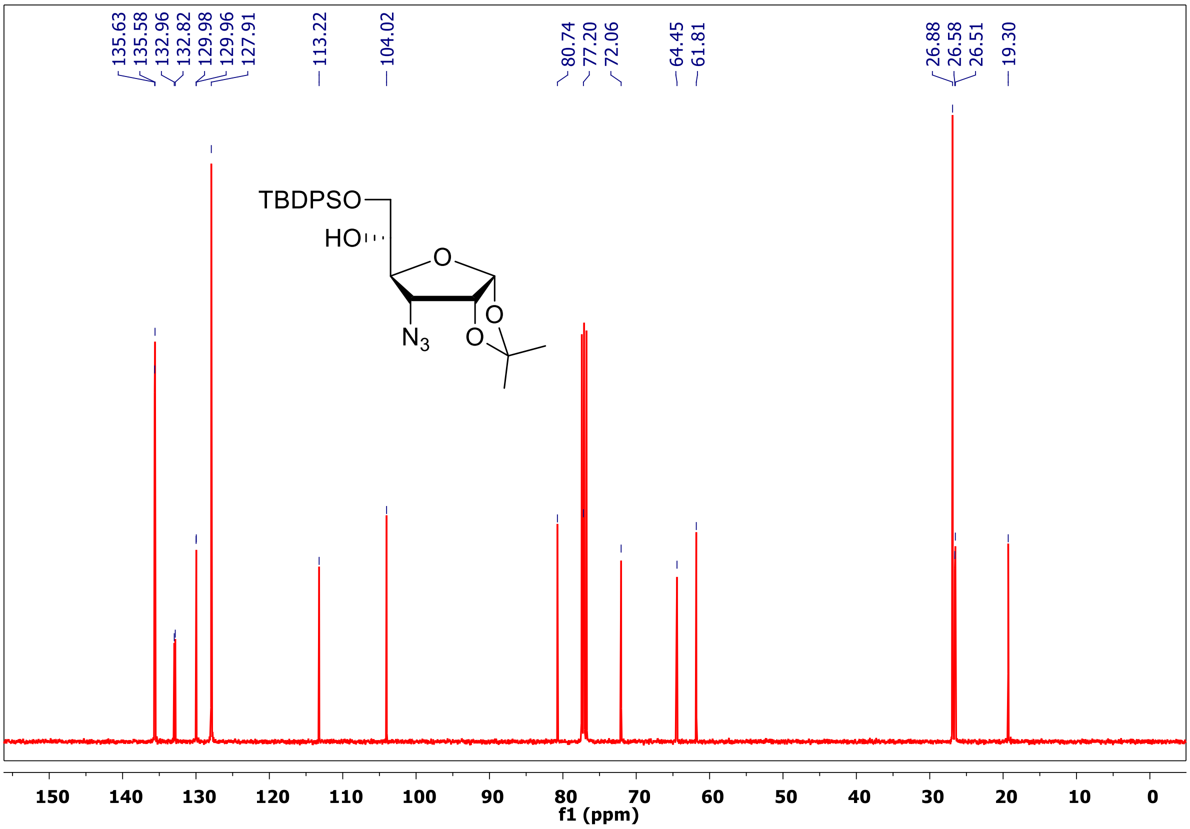

Figure S20: ^{13}C NMR spectrum of compound **18** (100.6 MHz, $CDCl_3$).

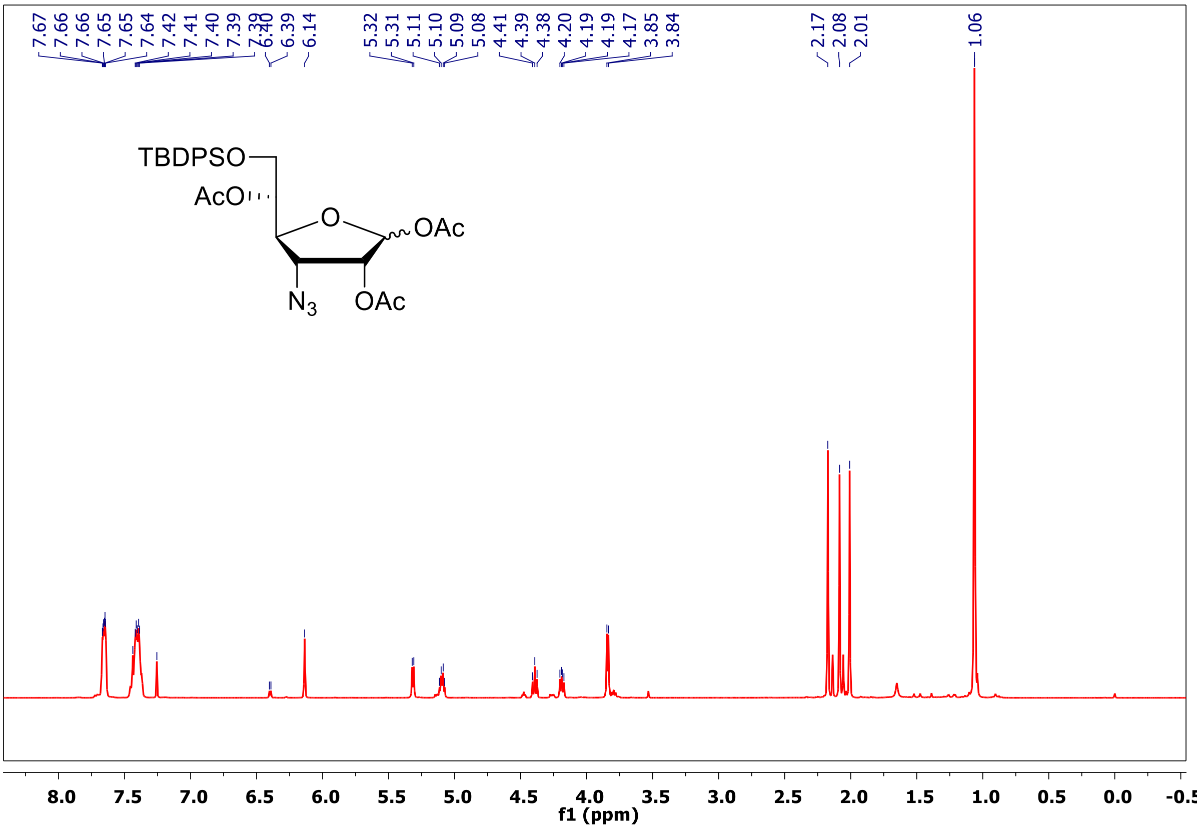

Figure S21: ^{1}H NMR spectrum of compound **19a, b** (400 MHz, $CDCl_3$).

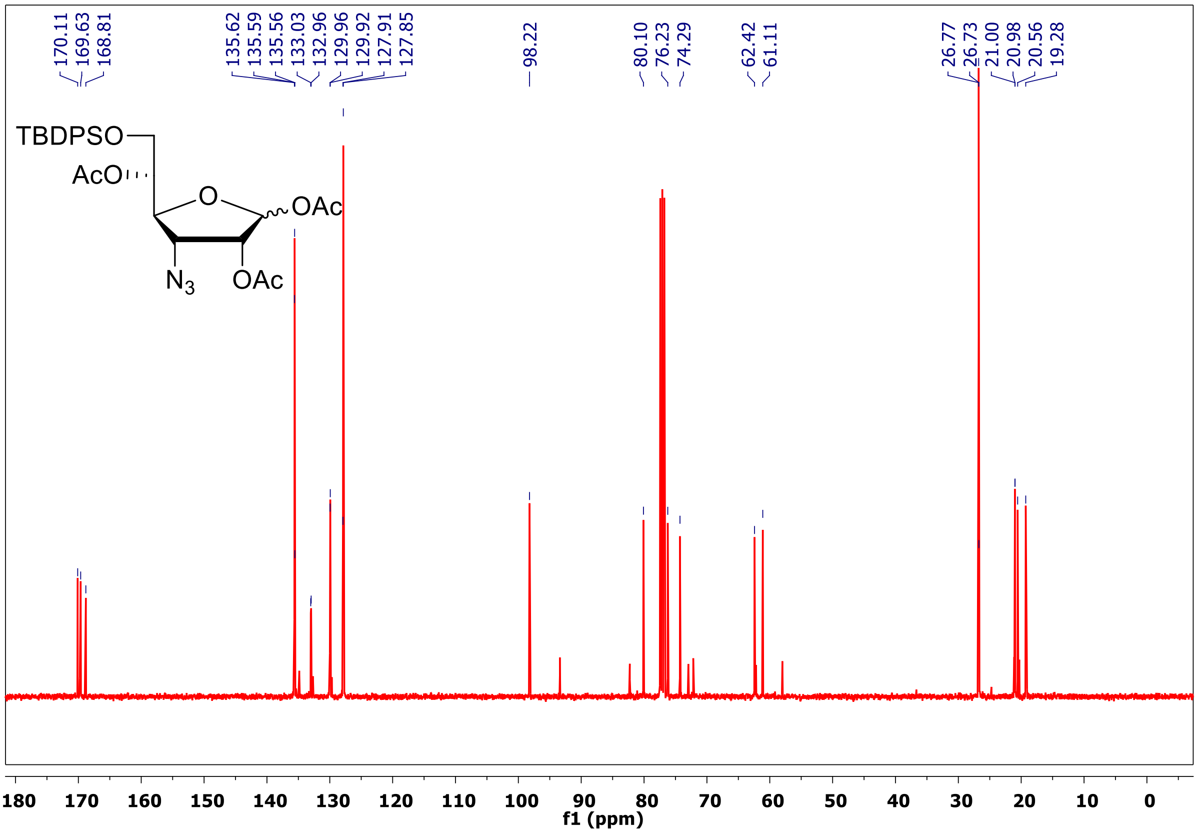

Figure S22: ^{13}C NMR spectrum of compound **19a, b** (100.6 MHz, $CDCl_3$).

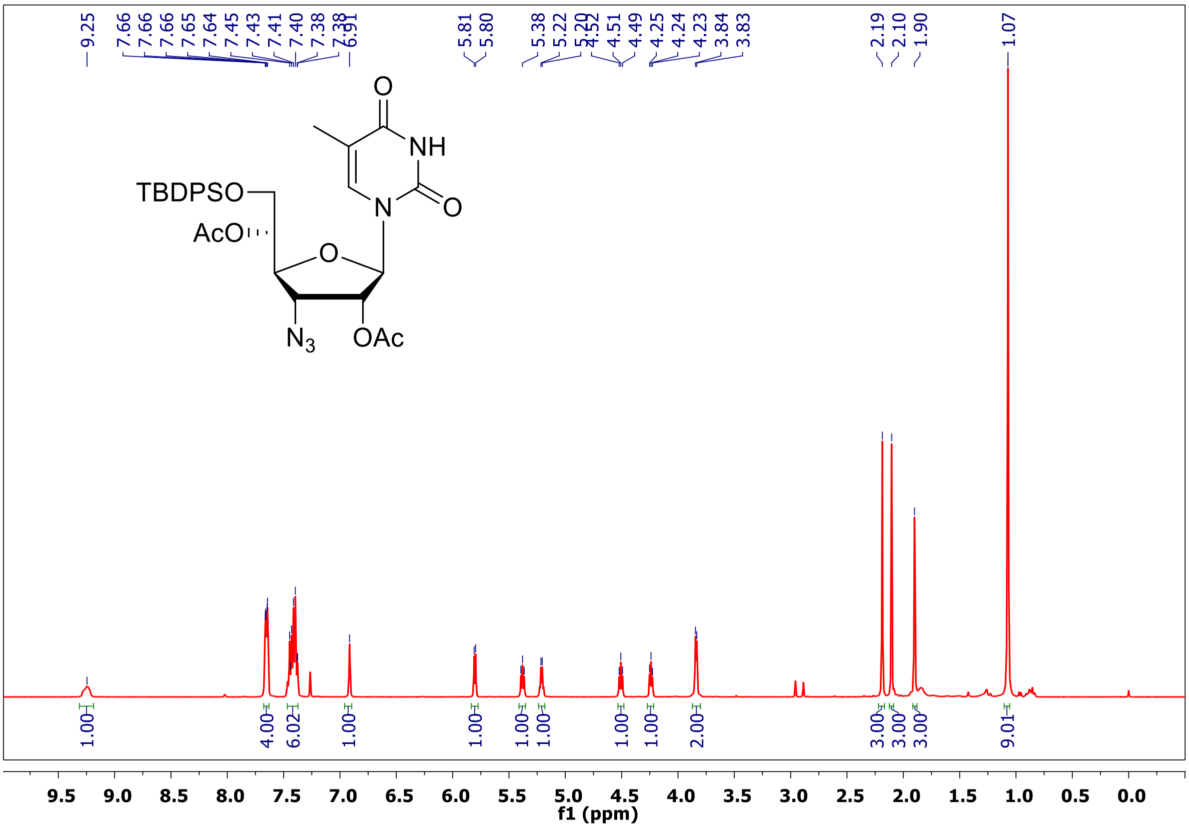

Figure S23: ^{1}H NMR spectrum of compound **20a** (400 MHz, $CDCl_3$).

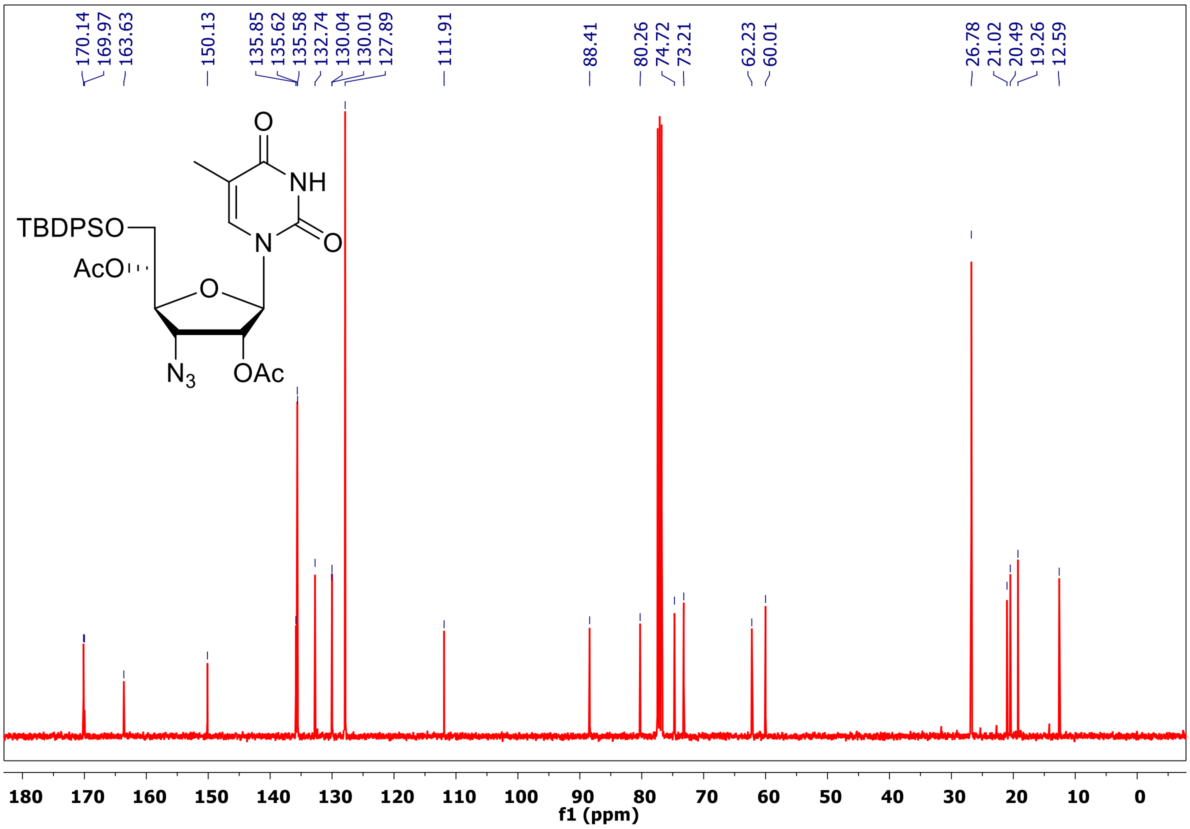

Figure S24: ^{13}C NMR spectrum of compound **20a** (100.6 MHz, $CDCl_3$).

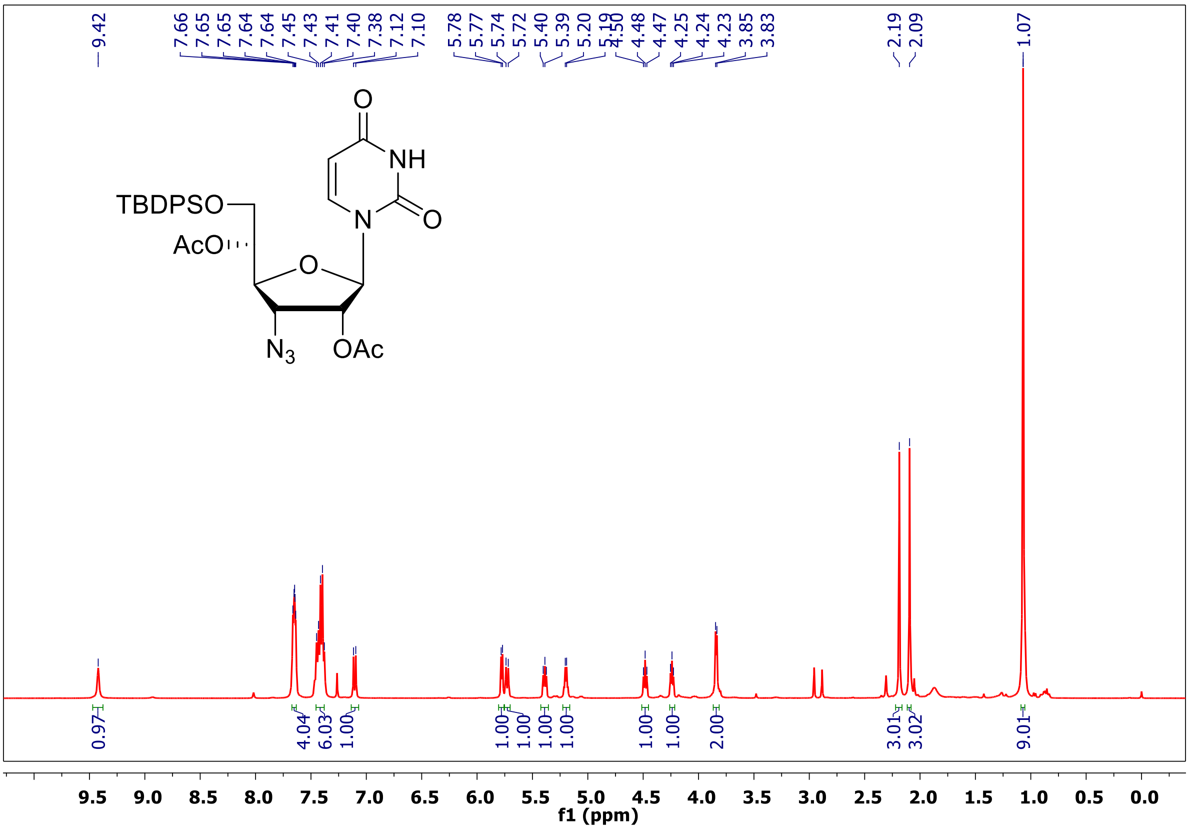

Figure S25: ^{1}H NMR spectrum of compound **20b** (400 MHz, $CDCl_3$).

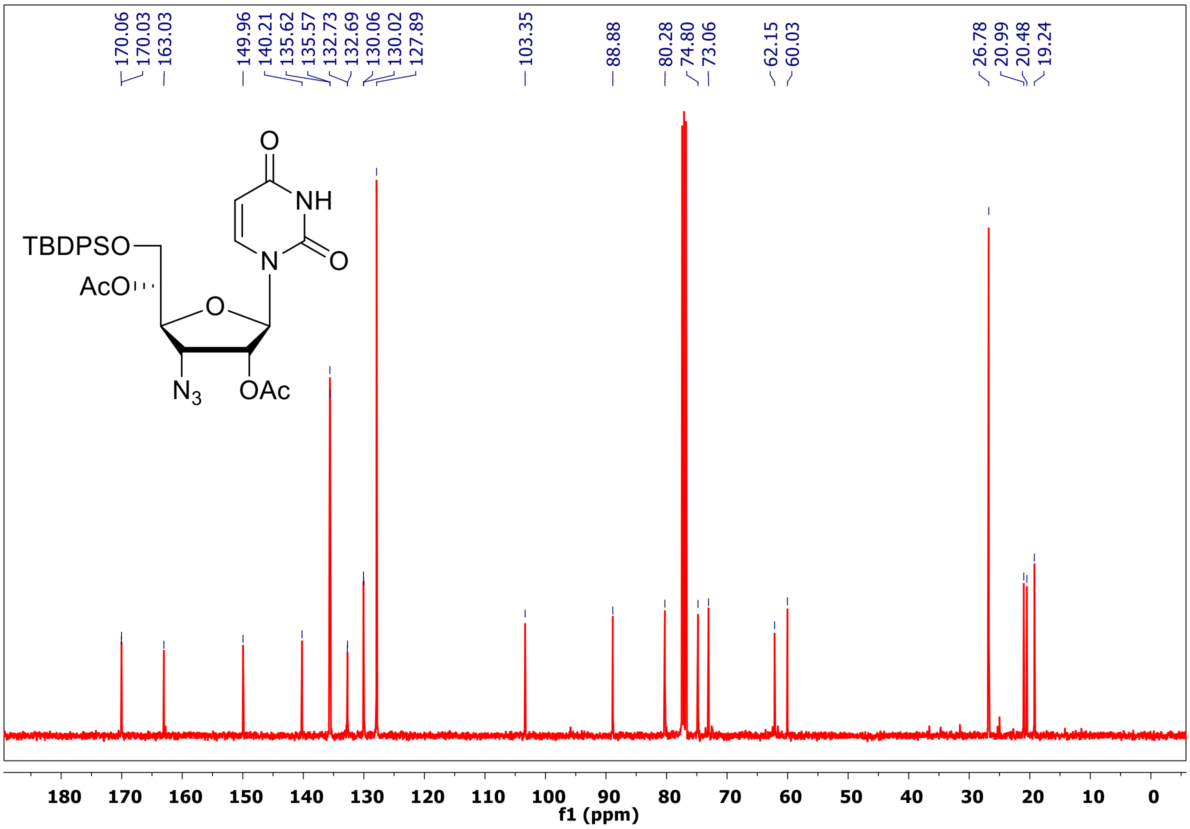

Figure S26: ^{13}C NMR spectrum of compound **20b** (100.6 MHz, $CDCl_3$).

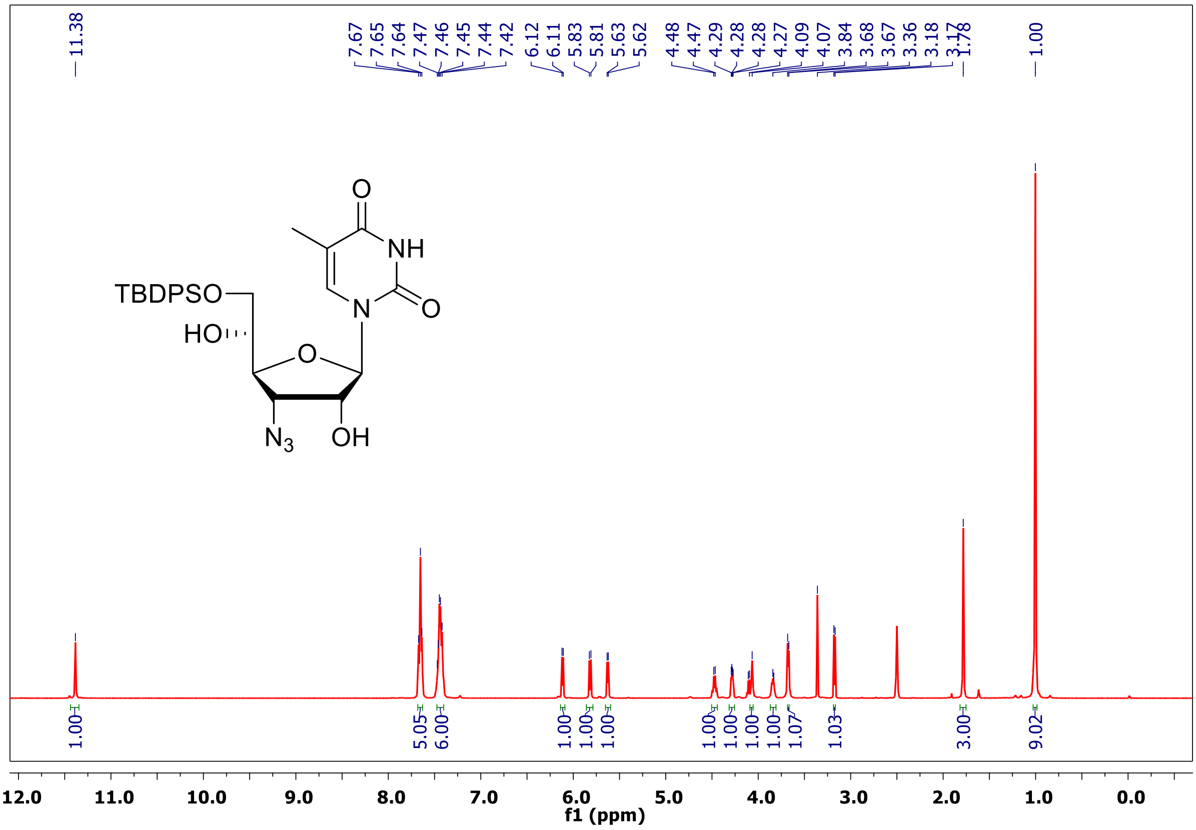

Figure S27: ^{1}H NMR spectrum of compound **21a** (400 MHz, DMSO-d_6).

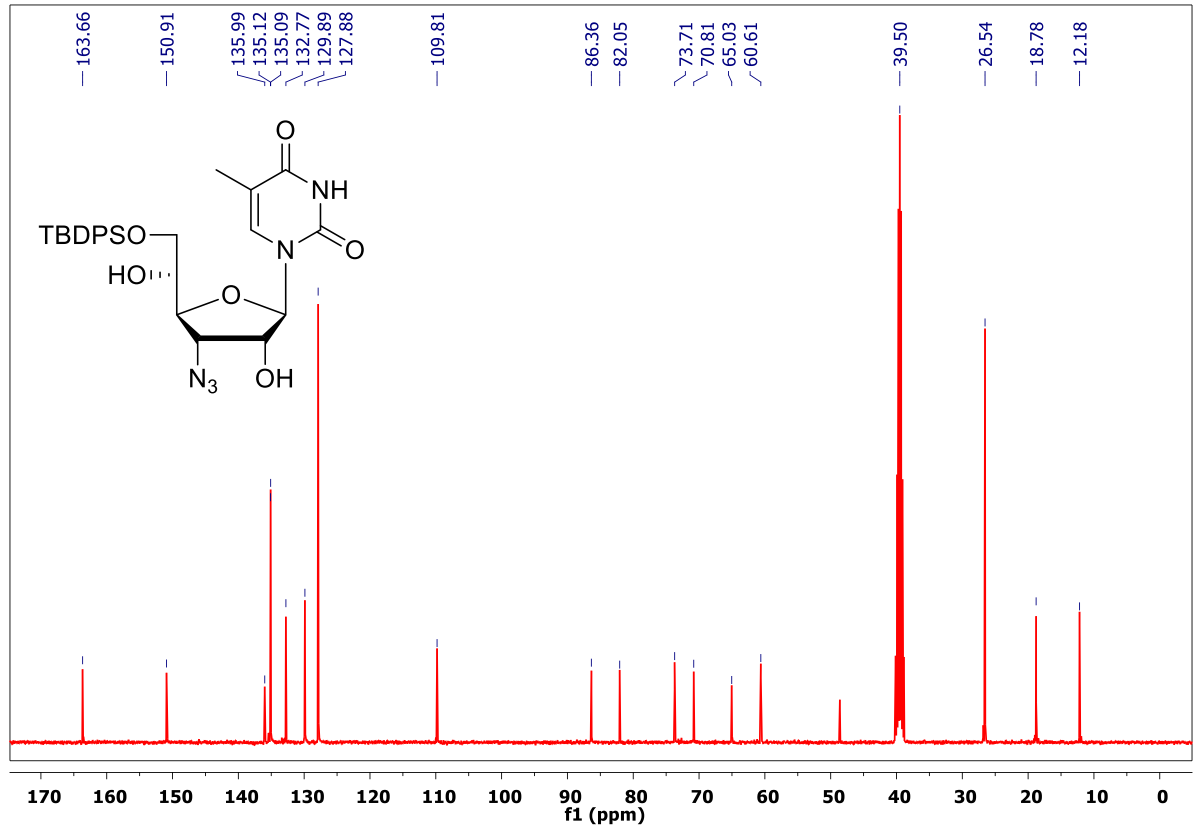

Figure S28: ^{13}C NMR spectrum of compound **21a** (100.6 MHz, DMSO-d_6).

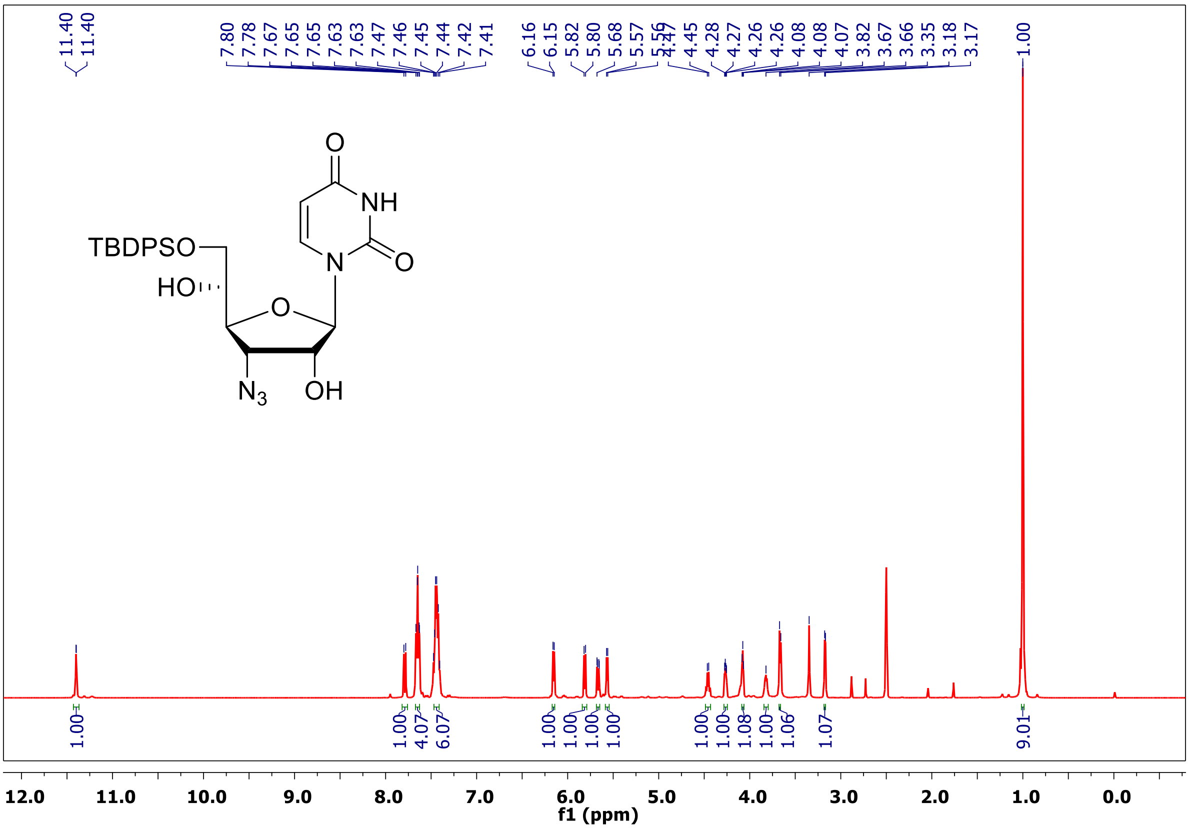

Figure S29: ^{1}H NMR spectrum of compound **21b** (400 MHz, DMSO-d_6).

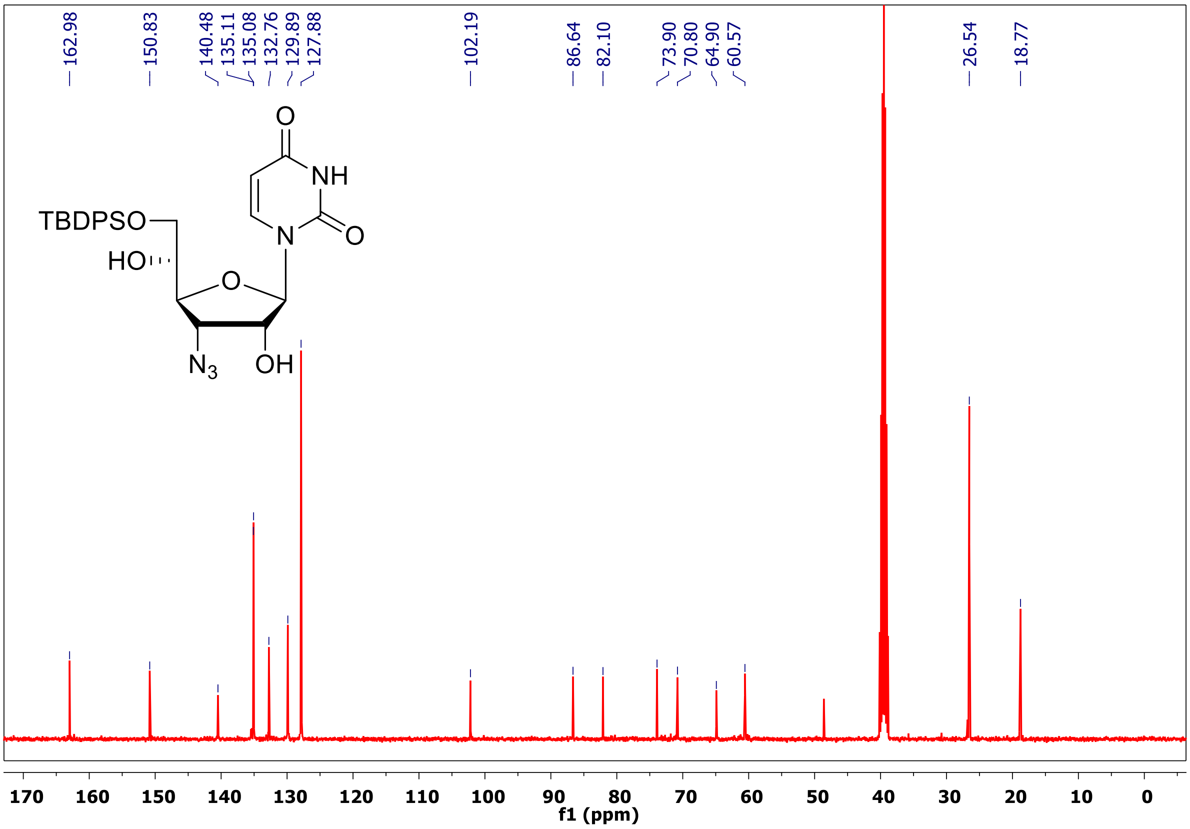

Figure S30: ^{13}C NMR spectrum of compound **21b** (100.6 MHz, DMSO-d_6).

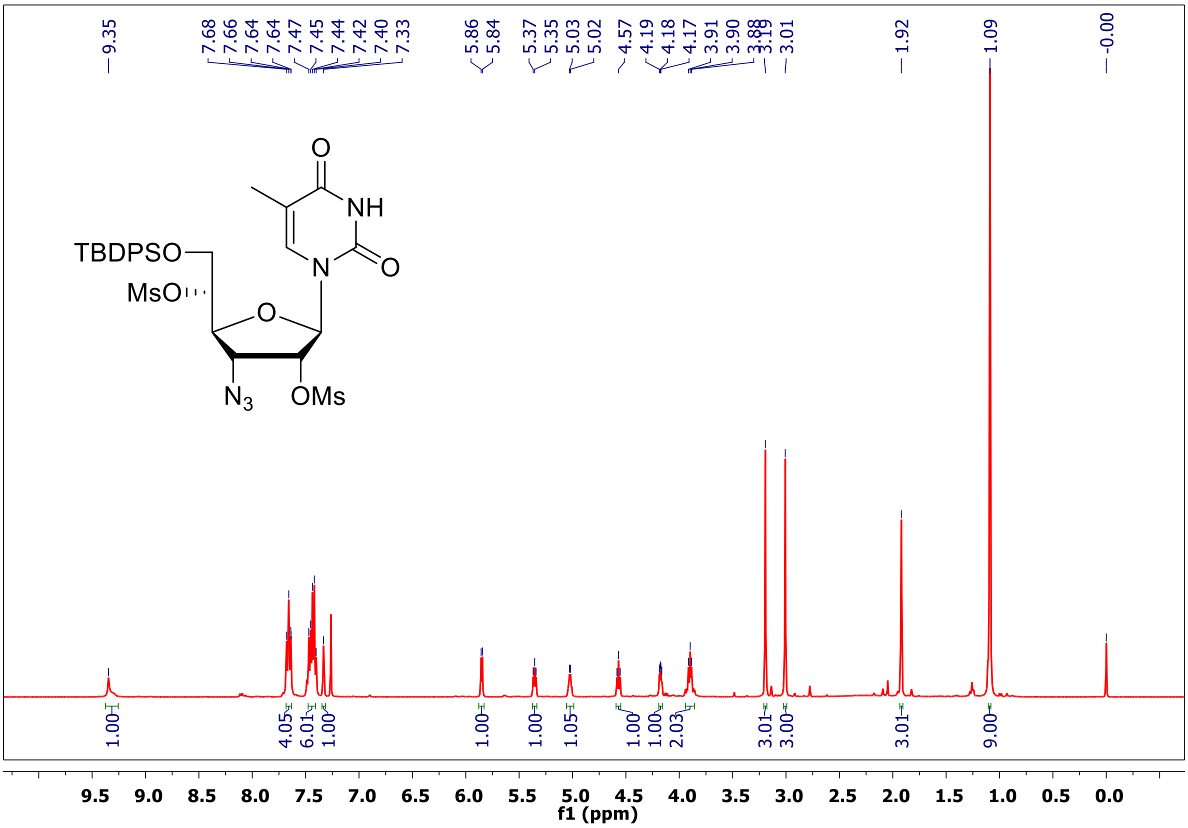

Figure S31: ^{1}H NMR spectrum of compound **22a** (400 MHz, $CDCl_3$).

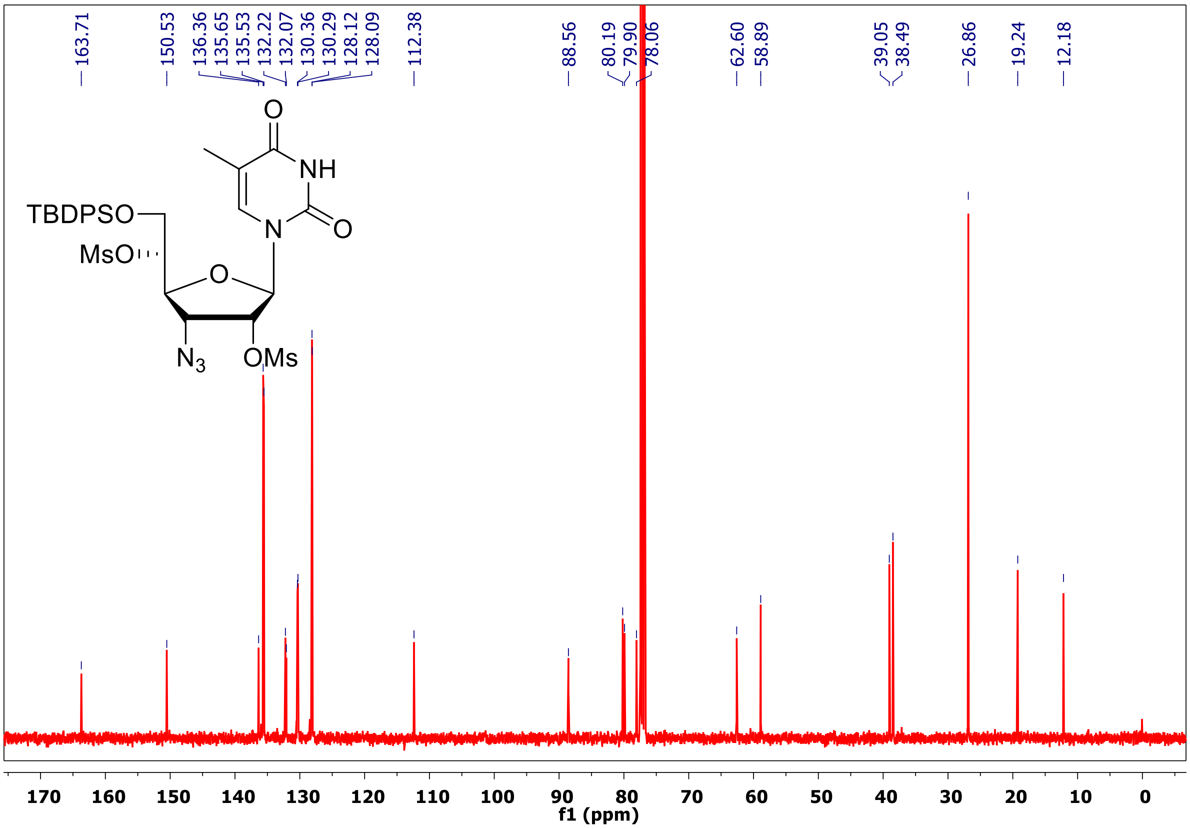

Figure S32: ^{13}C NMR spectrum of compound **22a** (100.6 MHz, $CDCl_3$).

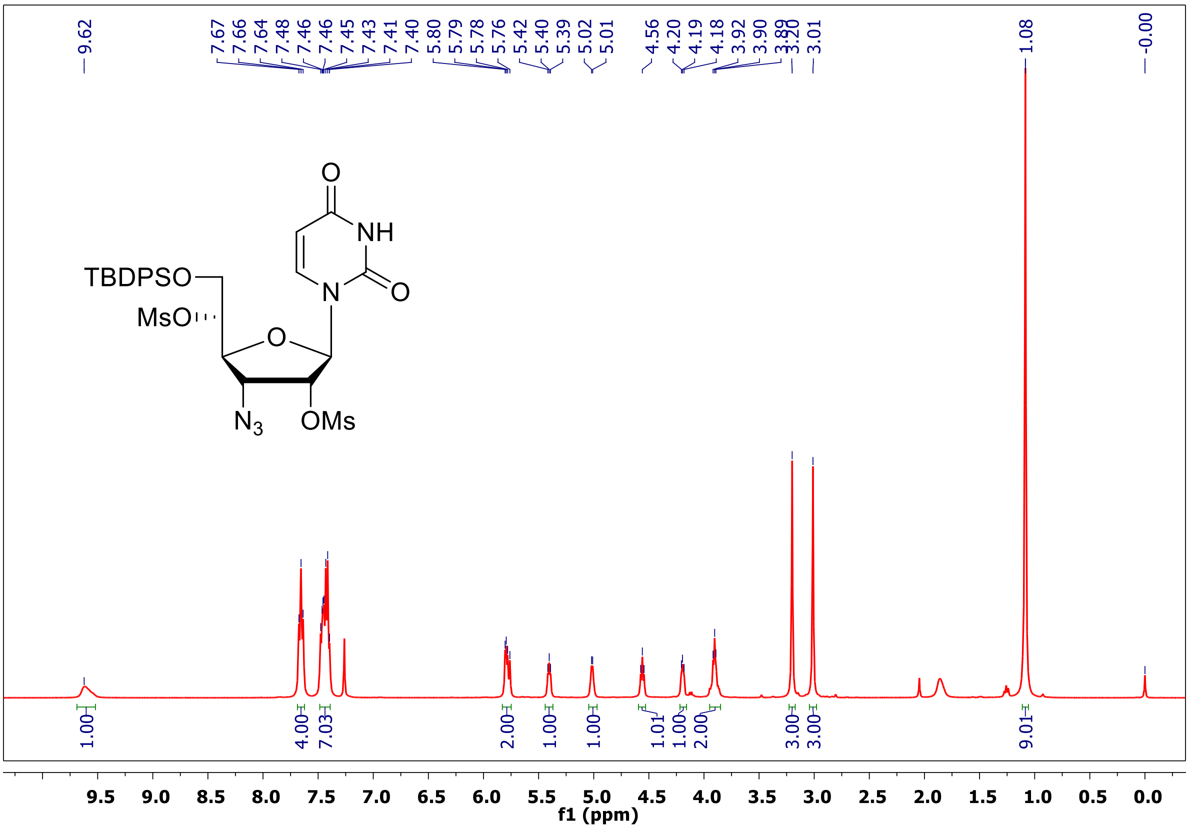

Figure S33: ^{1}H NMR spectrum of compound **22b** (400 MHz, $CDCl_3$).

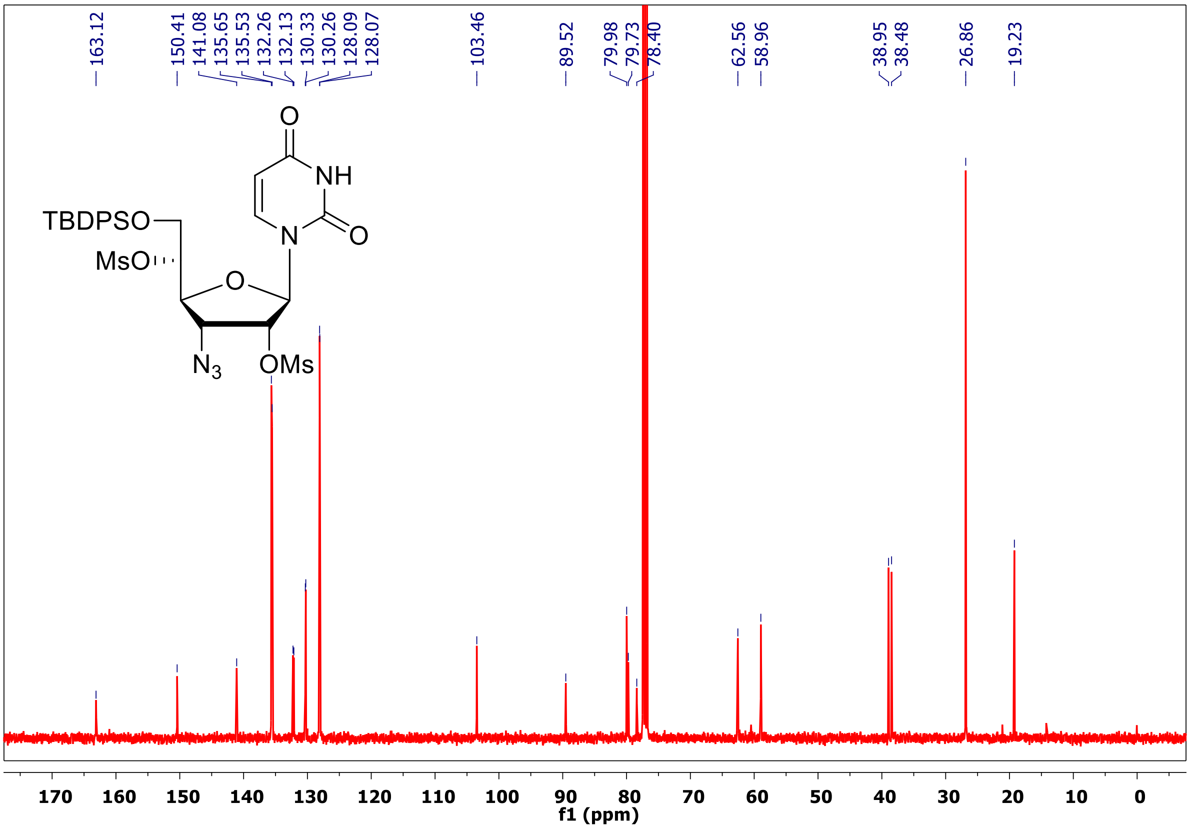

Figure S34: ^{13}C NMR spectrum of compound **22b** (100.6 MHz, $CDCl_3$).

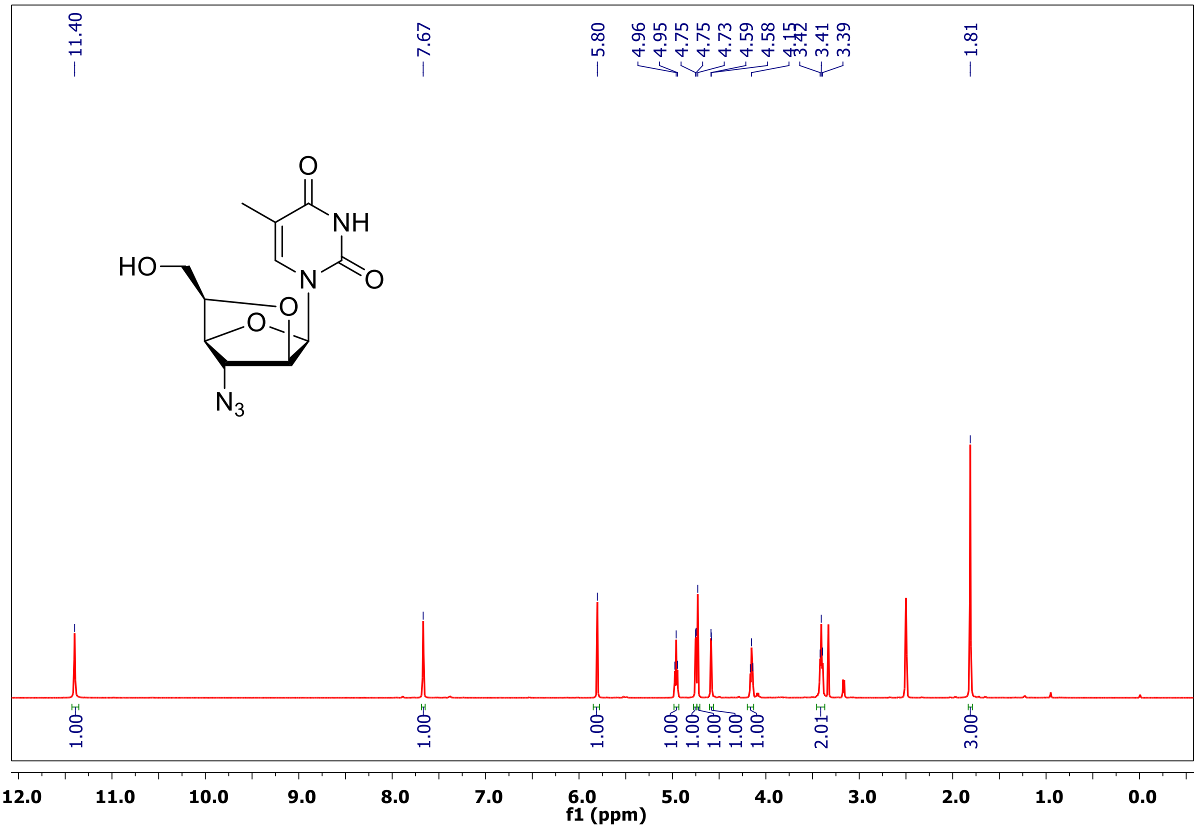

Figure S35: ^{1}H NMR spectrum of compound **9a** (400 MHz, DMSO-d_6).

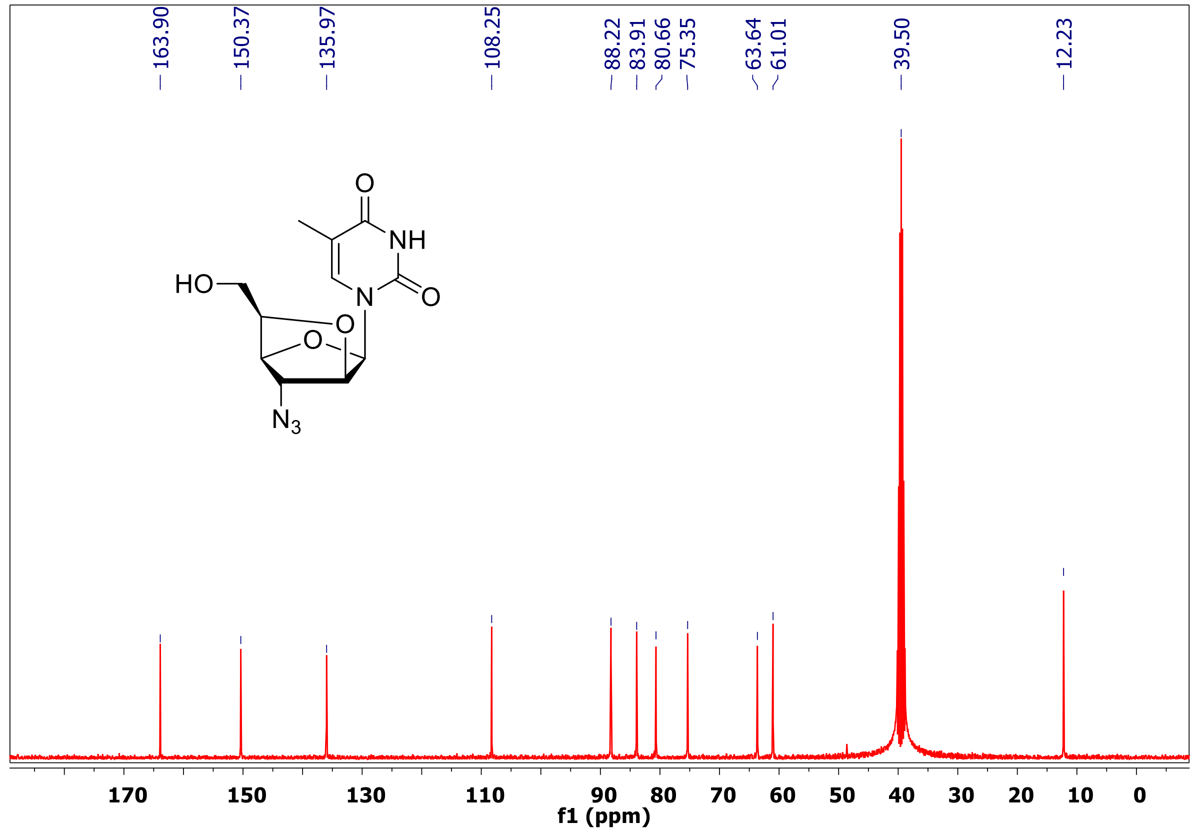

Figure S36: ^{13}C NMR spectrum of compound **9a** (100.6 MHz, DMSO-d_6).

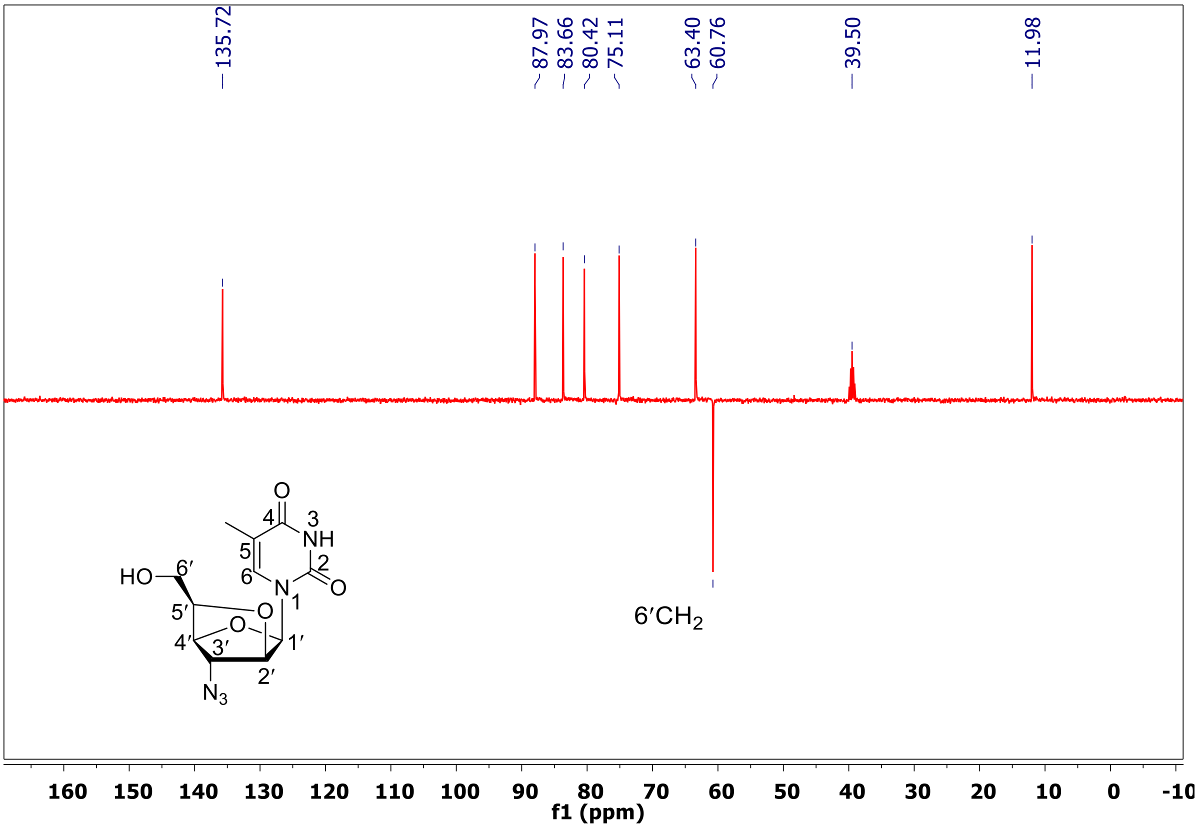

Figure S37: DEPT-135 NMR spectrum of compound **9a** (100.6 MHz, DMSO-d_6).

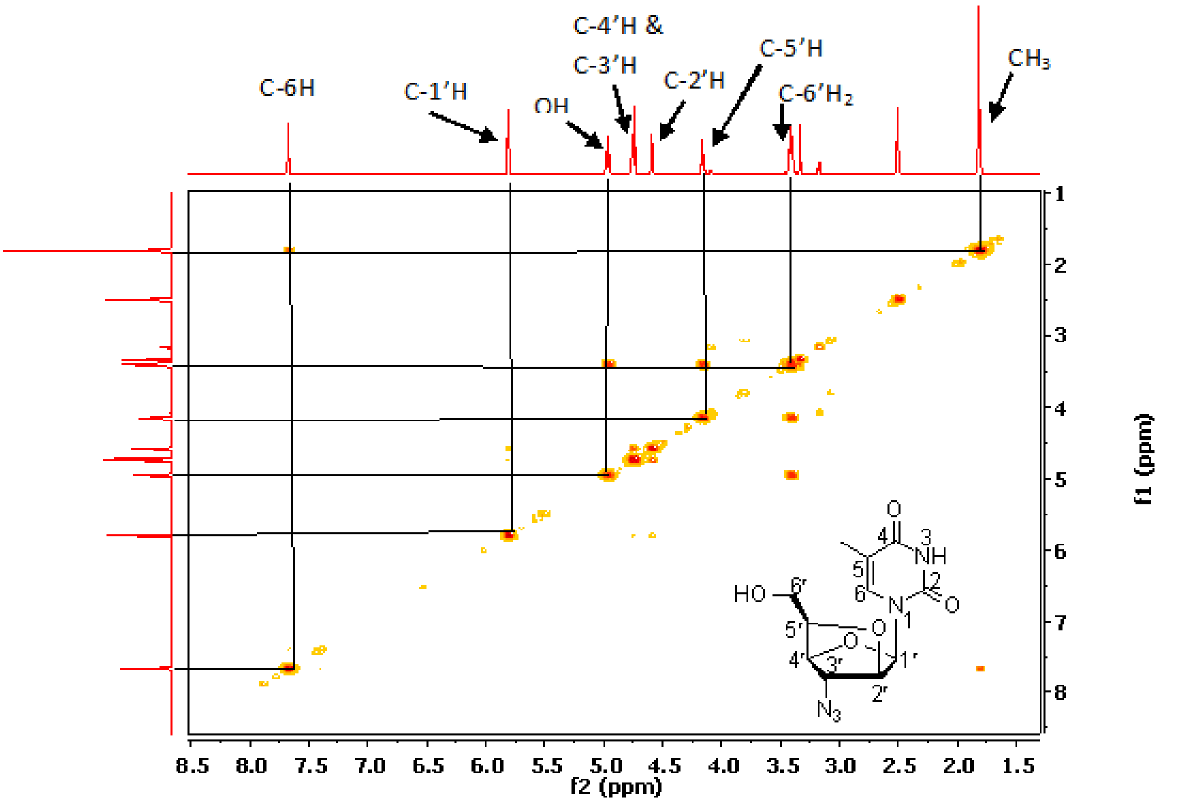

Figure S38: $^{1}H,^{1}H$ COSY NMR spectrum of compound **9a** (DMSO-d_6).

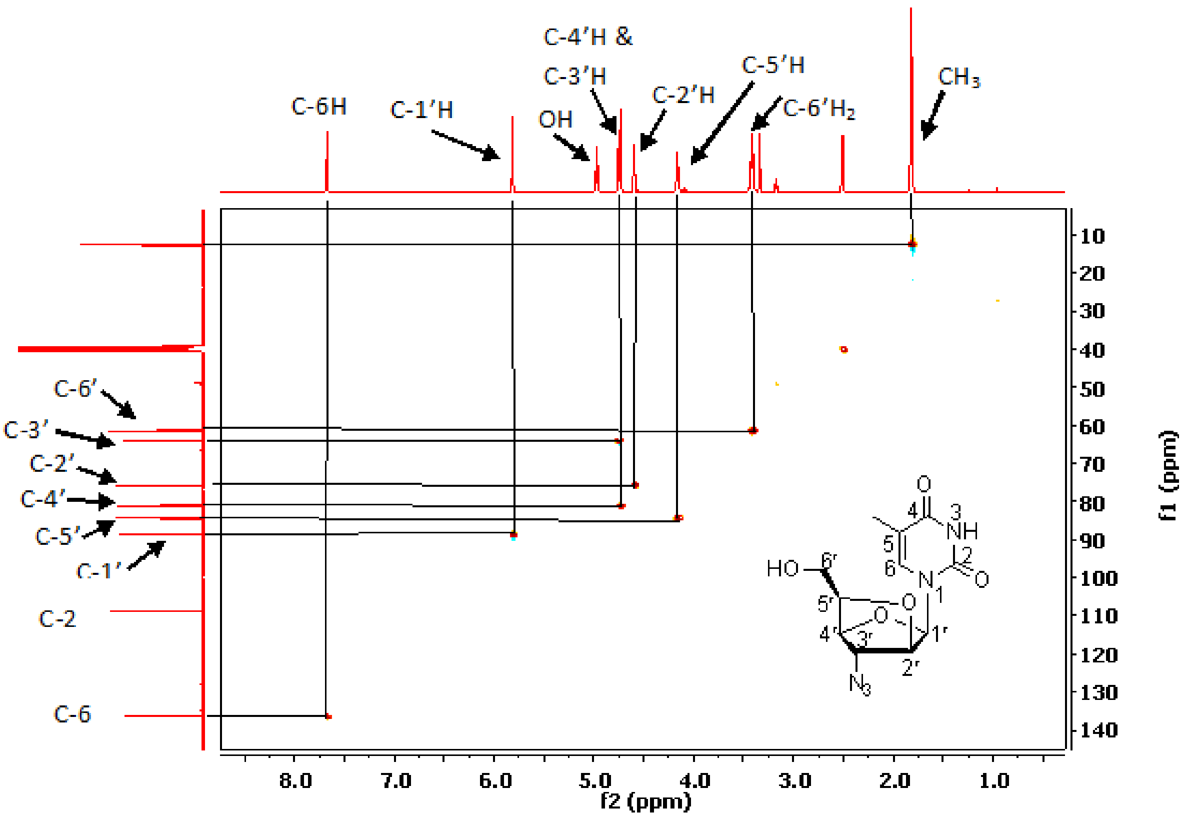

Figure S39: ^{1}H-^{13}C HETCOR NMR spectrum of compound **9a** (DMSO-d_6).

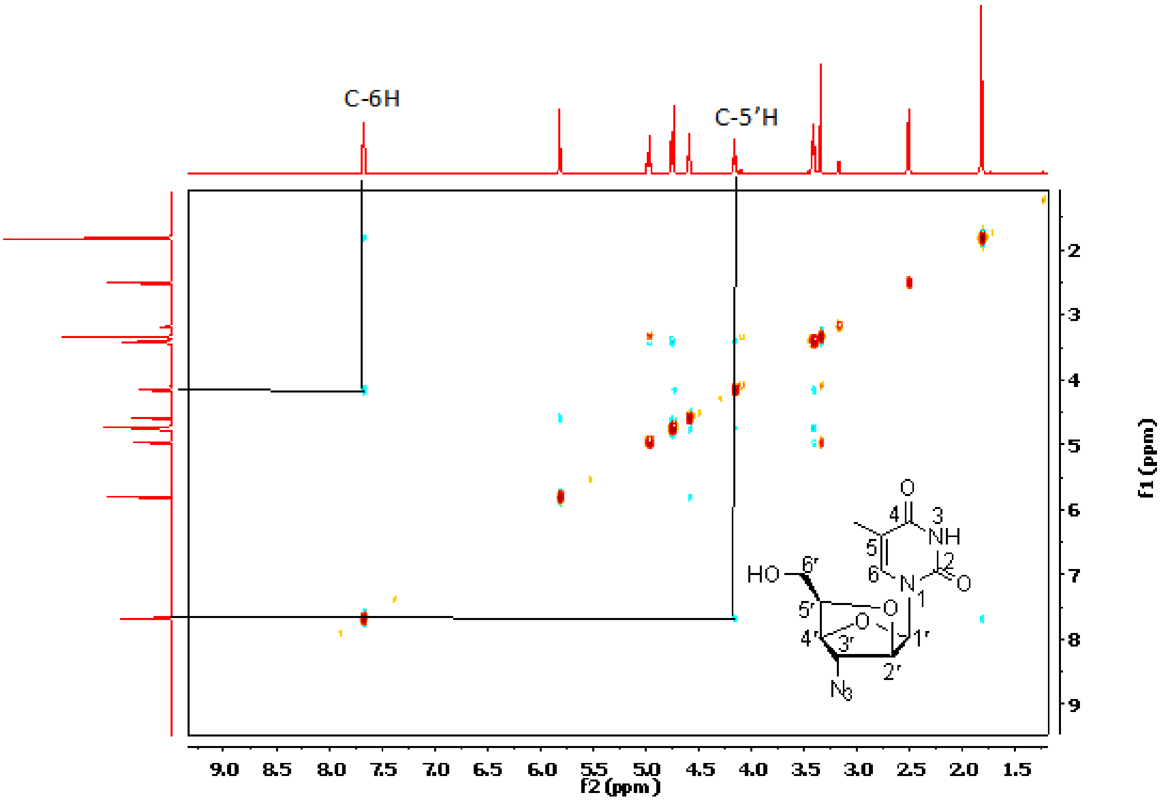

Figure S40: NOESY NMR spectrum of compound **9a**.

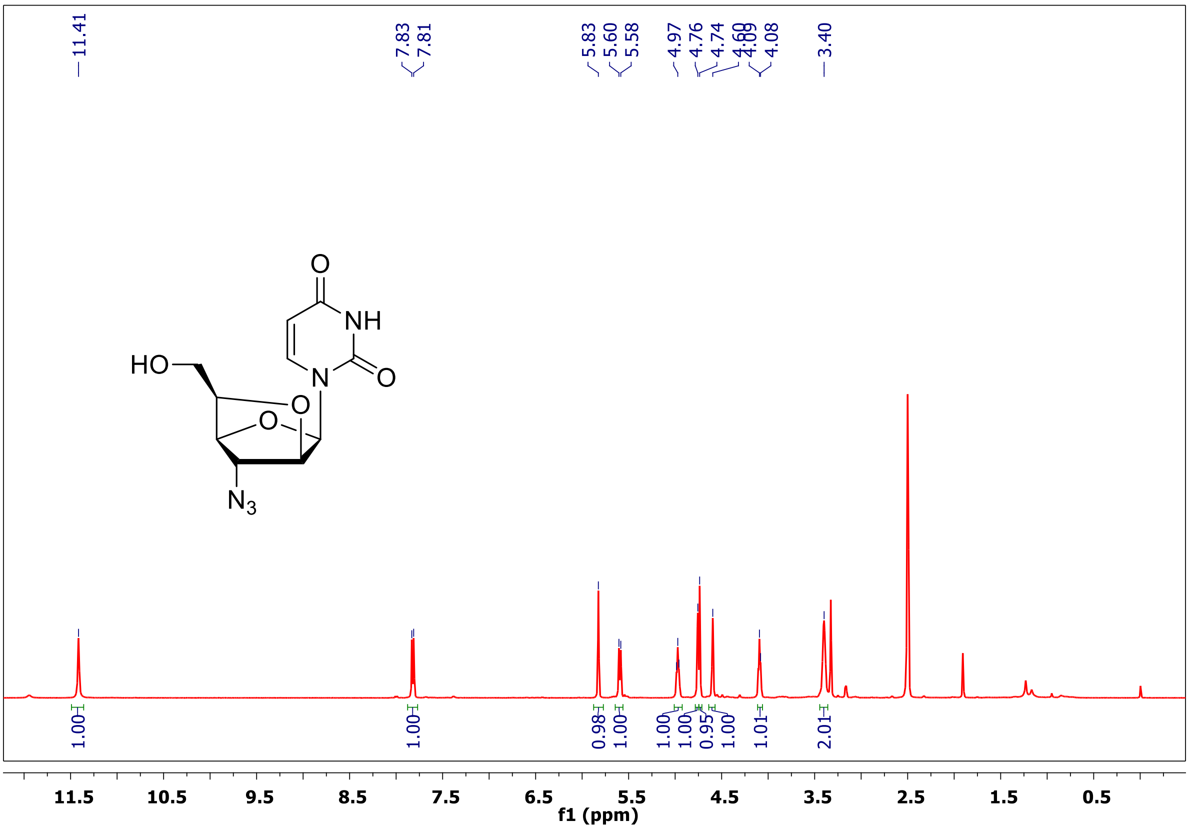

Figure S41: ^{1}H NMR spectrum of compound **9b** (400 MHz, DMSO-d_6).

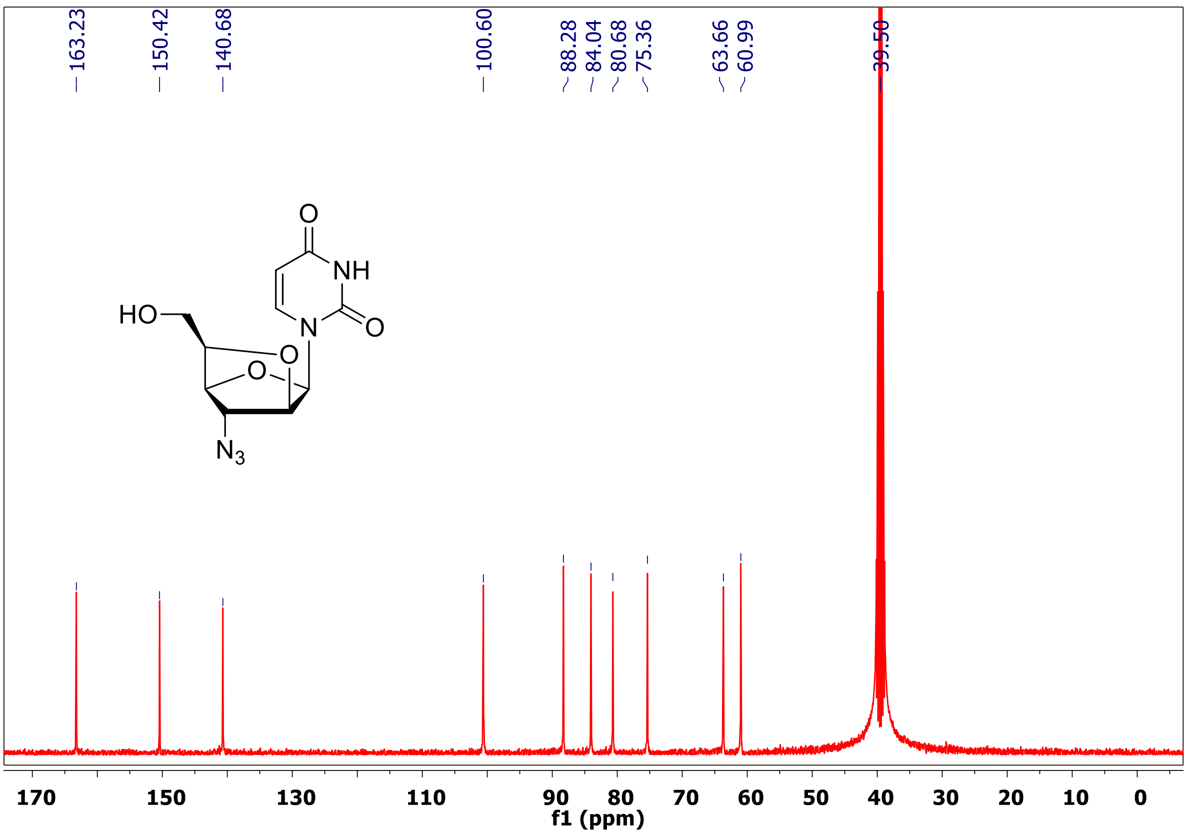

Figure S42: ^{13}C NMR spectrum of compound **9b** (100.6 MHz, DMSO-d_6).

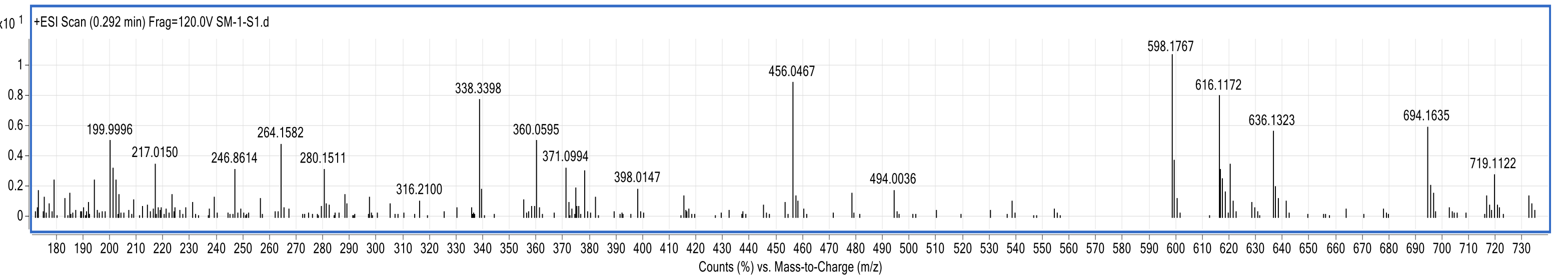

Figure S43. Mass spectrum of reaction mixture (conversion of **22b** to **9b**).
